# Supplementary material for: Host responses and viral traits interact to shape the impacts of climate warming on highly pathogenic avian influenza in migratory waterfowl
Source: PLoS Comput Biol. 2025 Oct 6;21(10):e1013451. doi: 10.1371/journal.pcbi.1013451 (PMC12513652; doi:10.1371/journal.pcbi.1013451)
Supplement: S1 Text — (DOCX) [file pcbi.1013451.s001.docx]

**Host responses and viral traits interact to shape the impacts of climate warming on highly pathogenic avian influenza in migratory waterfowl**

Claire S. Teitelbaum, Michael L. Casazza, Cory T. Overton, Elliott L. Matchett, Diann J. Prosser

# Supplementary methods

## Equations

This model includes multiple hosts and multiple subtypes. The model is based on models described by Brown *et al.* [1]. We make several key modifications to these models:

1. Cross-immunity is symmetric: recovery from infection with strain 1 (LPAI) provides the same protection against infection by strain 2 (HPAI) as HPAI does for LPAI.
2. Strain 2 (HPAI) causes mortality at rate $\nu_{2}$.
3. A second cross-immunity parameter ($\psi_{2}$) modifies this mortality rate in individuals previously infected by LPAI.
4. Virions also enter the environment at a constant rate ($\zeta_{2}$), representing shedding of heterospecifics (or conspecifics from another sub-population).

The full model operates as follows:

Individuals susceptible to both strains (class $N_{SS}$) are born at rate $b(t)$, which is nonzero only at the breeding site and during the breeding season (0.38 < *t* < 0.76). Infections with strain 1 (LPAI) occur with the force of infection rate $\lambda_{1}$, and infections with strain 2 (HPAI) occur at rate $\lambda_{2}$. Individuals can also enter class $N_{SS}$ via waning immunity at rates $\epsilon_{1}$ (LPAI) or $\epsilon_{2}$ (HPAI). Natural mortality occurs for all infection classes at rate $\mu$, which is constant across time except at the breeding site, where it is elevated during winter (i.e., birds do not survive on Alaskan breeding grounds in winter).

$$\frac{dN_{SS}}{dt}=b(t)N-(\lambda_{1}+\lambda_{2})N_{SS}+\epsilon_{1}N_{RS}+\epsilon_{2}N_{SR}-\mu(t)N_{SS}$$

In the equation above, $\lambda_{i}$ is the transmission rate for strain $i$, incorporating both direct and indirect transmission. $\beta_{i}$ is the rate of transmission from contact (which varies across sites), $\delta_{i}$ is the relative abundance of each viral strain in the environment (see below), $\rho$ is the uptake rate of virions from the environment, $\kappa_{i}$ is the infectious dose (ID_50_) of a given strain, $V_{i}$ is the amount of virus in the environment:

$$\lambda_{i}=\beta_{i}I_{i}+\delta_{i}\rho\left( \frac{\rho V_{i}}{\rho V_{i}+\kappa_{i}} \right)V_{i}$$

$\delta_{i}$ is the relative force of infection for each strain in the environment (i.e., controls which strain is more likely to be encountered in the environment) and is scaled by the infectious dose of the strain in question:

$$\delta_{i}=\frac{V_{i}/\kappa_{i}}{V_{1}/\kappa_{1}+V_{2}/\kappa_{2}}$$

This scaling effectively represents the number of infectious doses a bird is likely to encounter. Birds infected with LPAI but susceptible to HPAI ($N_{IS}$) enter this class from the fully susceptible category via infection at rate $\lambda_{1}$, or via waning immunity from the group of birds that are infected with LPAI and recovered from HPAI (at rate $\epsilon_{2})$. Individuals leave this class if they become infected with HPAI (at rate $\lambda_{2}$, modified by the cross-immunity parameter $\psi_{1}$), through recovery from infection at rate $\gamma_{1}$, or through natural mortality.

$$\frac{dN_{IS}}{dt}=\lambda_{1}N_{SS}+\epsilon_{2}N_{IR}-(1-\psi_{1})\lambda_{2}N_{IS}-(\gamma_{1}+\mu)N_{IS}$$

The same processes apply to birds infected with HPAI but susceptible to LPAI ($N_{SI}$). There is additional disease-induced mortality from HPAI at rate $\nu_{2}$.

$$\frac{dN_{SI}}{dt}=\lambda_{2}N_{SS}+\epsilon_{1}N_{RI}-(1-\psi_{1})\lambda_{1}N_{SI}-(\gamma_{2}+\mu+\nu_{2})N_{SI}$$

Birds can be simultaneously infected with both strains $\left( N_{II} \right)$. Birds enter this class via infection at rates $(1-\psi_{1})\lambda_{2}$ and $(1-\psi_{1})\lambda_{1}$, and leave the class via recovery from infection ($\gamma_{2}$ and $\gamma_{1}$) or mortality (natural, $\mu$, or disease-induced, $\nu_{2}$).

$$\frac{dN_{II}}{dt}=(1-\psi_{1})\lambda_{2}N_{IS}+(1-\psi_{1})\lambda_{1}N_{SI}-(\gamma_{2}+\gamma_{1}+\mu+\nu_{2})N_{II}$$

Birds infected with LPAI and recovered from HPAI ($N_{IR}$) enter this class through recovery from HPAI after being co-infected with both strains (at rate $\gamma_{2}$) or if they become infected with LPAI after having recovered from HPAI (at the cross-immunity-modified transmission rate $(1-\psi_{1})\lambda_{1}$). They leave this class through recovery from LPAI infection (at rate $\gamma_{1}$), loss of immunity to HPAI (at rate $\epsilon_{2}$), or natural mortality ($\mu$).

$$\frac{dN_{IR}}{dt}=\gamma_{2}N_{II}+(1-\psi_{1})\lambda_{1}N_{SR}-(\gamma_{1}+\mu+\epsilon_{2})N_{IR}$$

Birds recovered from LPAI and infected with HPAI ($N_{RI}$) enter this class through recovery from LPAI after being co-infected with both strains (at rate $\gamma_{1}$) or if they become infected with HPAI after having recovered from LPAI (at the cross-immunity-modified transmission rate $(1-\psi_{1})\lambda_{2}$). They leave this class through recovery from HPAI infection (at rate $\gamma_{2}$), loss of immunity to LPAI (at rate $\epsilon_{1}$), natural mortality ($\mu$), or disease-induced mortality; the rate of disease-induced mortality is modified by the cross-protection parameter $\psi_{2}$.

$$\frac{dN_{RI}}{dt}=\gamma_{1}N_{II}+(1-\psi_{1})\lambda_{2}N_{RS}-(\gamma_{2}+\mu+\epsilon_{1}+(1-\psi_{2})\nu_{2})N_{RI}$$

Birds susceptible to HPAI and recovered from LPAI ($N_{RS}$) enter this class through recovery from LPAI (at rate $\gamma_{1}$) or through waning immunity from the class of birds recovered from both strains (at rate $\epsilon_{2}$). Birds leave this class through infection with HPAI, at the cross-immunity-modified transmission rate $(1-\psi_{1})\lambda_{2}$. They can also leave the class through waning immunity from LPAI at rate $\epsilon_{1}$ and through natural mortality.

$$\frac{dN_{RS}}{dt}=\gamma_{1}N_{IS}+\epsilon_{2}N_{RR}-\left( 1-\psi_{1} \right)\lambda_{2}N_{RS}-\left( \epsilon_{1}+\mu\right)N_{RS}$$

The same processes apply to birds susceptible to LPAI and recovered from HPAI ($N_{SR}$).

$$\frac{dN_{SR}}{dt}=\gamma_{2}N_{SI}+\epsilon_{1}N_{RR}-(1-\psi_{1})\lambda_{1}N_{SR}-(\epsilon_{1}+\mu)N_{SR}$$

Birds that are simultaneously recovered from both strains ($N_{RR}$) enter this class via recovery from one while already recovered from the other (at rates $\gamma_{1}$ and $\gamma_{2}$) and leave the category through waning immunity.

$$\frac{dN_{RR}}{dt}=\gamma_{1}N_{IR}+\gamma_{2}N_{RI}-(\epsilon_{1}+\epsilon_{2})N_{RR}$$

Finally, virions enter the environment via shedding from infected individuals at a strain-specific shedding rate ($\omega_{1}$ or $\omega_{2}$), and decay at a time-varying and strain-specific rate $\eta_{1}(T)$or $\eta_{2}(T)$, which is temperature (T)-dependent. Virions also enter the environment via shedding from infected heterospecifics at a constant rate $\zeta_{i}$. $I_{1}$ is the total number of individuals infected with LPAI (i.e., $N_{IS}+N_{II}+N_{IR}$).

$$\frac{dV_{i}}{dt}=\omega_{i}I_{i}-\eta_{i}(T)V_{i}+\zeta_{i}$$

## Animal telemetry data analysis

Goose telemetry data were derived from the data set described by Overton and Casazza [2]. Geese used in this study were captured and fit with GPS transmitters between 2016 and 2022. For each bird, we plotted net displacement (i.e., distance from first location) over time to identify winter, breeding, migration, and stopover periods. We extracted the first and last date of each season for each individual in each year to parameterize the range of seasonal dates in the models.

Next, we identified the primary spatial location of each seasonal site, which was later used to extract climate data for each site. For winter and breeding seasons, we defined the sites as the 99% contour of the kernel density estimate (KDE) of all locations with that seasonal classification. For fall and spring migrations, we used known stopover locations for Pacific Flyway greater white-fronted geese: southern Alaska (the Paimut Slough, Cook Inlet, and Copper River Delta) in fall and the Southern Oregon and Northern California (SONEC) region in spring [3–5]. We defined the fall stopover area as the 95% minimum convex polygon (MCP) of all GPS locations assigned the “fall stopover” class near the Paimut Slough/Cook Inlet (bounding box: 166°W, 140°W 55°N, 65°N). Similarly, we defined the spring stopover area as the 95% minimum convex polygon of all spring stopover locations in SONEC (bounding box: 122.5°W, 120°W, 40.5°N, 43°N). We preferred using MCPs rather than KDEs for stopover seasons because some geese used additional or alternative stopover sites that were not included in the four-site model framework. MCPs and KDEs were calculated using the *adehabitatHR* and *amt* packages in R [6–8].

Any use of trade, firm, or product names is for descriptive purposes only and does not imply endorsement by the U.S. Government.

## Bibliography

1. Brown VL, Drake JM, Barton HD, Stallknecht DE, Brown JD, Rohani P. Neutrality, cross-immunity and subtype dominance in avian influenza viruses. PLoS ONE. 2014;9. doi:10.1371/journal.pone.0088817

2. Overton CT, Casazza ML. Movement behavior, habitat selection, and functional responses to habitat availability among four species of wintering waterfowl in California. Front Ecol Evol. 2023;11: 1232704. doi:10.3389/fevo.2023.1232704

3. Ely CR, Takekawa JY. Geographic Variation in Migratory Behavior of Greater White-Fronted Geese (Anser Albifrons). The Auk. 1996;113: 889–901. doi:10.2307/4088866

4. Fleskes JP, Yee JL. Waterfowl distribution and abundance during spring migration in Southern Oregon and Northeastern California. Western North American Naturalist. 2007;67: 409–428. doi:10.3398/1527-0904(2007)67%5B409:WDAADS%5D2.0.CO;2

5. Pacific Flyway Council. Pacific Flyway management plan for the greater white-fronted goose. Portland, OR: Pacific Flyway Council, U.S. Fish and Wildlife Service, Canadian Wildlife Service, Dirección General de Conservación Ecológica de Recursos Naturales; 2003 July p. 27.

6. Calenge C, Fortmann-Roe S. adehabitatHR: Home Range Estimation. 2011. p. 0.4.21. doi:10.32614/CRAN.package.adehabitatHR

7. R Development Core Team. R: A Language and Environment for Statistical Computing. Vienna, Austria: R Foundation for Statistical Computing; 2023. Available: http://www.r-project.org

8. Signer J, Fieberg J, Avgar T. Animal movement tools (amt): R package for managing tracking data and conducting habitat selection analyses. Ecology and Evolution. 2019;9: 880–890. doi:10.1002/ece3.4823
