# Supplementary material for: Host responses and viral traits interact to shape the impacts of climate warming on highly pathogenic avian influenza in migratory waterfowl
Source: PLoS Comput Biol. 2025 Oct 6;21(10):e1013451. doi: 10.1371/journal.pcbi.1013451 (PMC12513652; doi:10.1371/journal.pcbi.1013451)
Supplement: S1 Fig — See main methods for more details on data inputs, simulation methods, and analysis methods. (PDF) [file pcbi.1013451.s002.pdf]

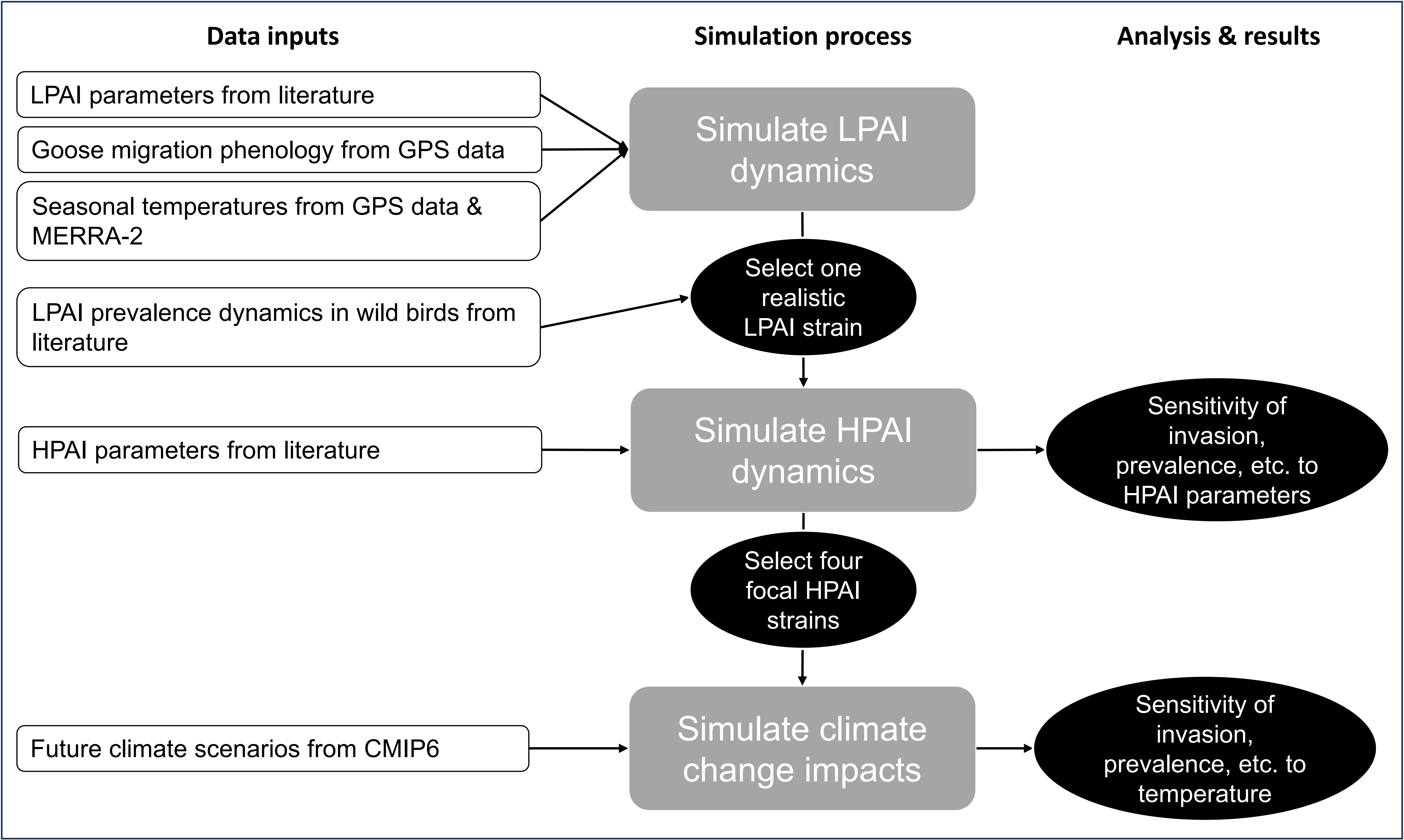
Data inputs
Simulation process
Analysis & results
LPAI parameters from literature
Goose migration phenology from GPS data
Seasonal temperatures from GPS data & MERRA-2
LPAI prevalence dynamics in wild birds from literature
HPAI parameters from literature
Future climate scenarios from CMIP6
Simulate LPAI dynamics
Select one realistic LPAI strain
Simulate HPAI dynamics
Select four focal HPAI strains
Simulate climate change impacts
Sensitivity of invasion, prevalence, etc. to HPAI parameters
Sensitivity of invasion, prevalence, etc. to temperature
