# Supplementary figures and images for: Host responses and viral traits interact to shape the impacts of climate warming on highly pathogenic avian influenza in migratory waterfowl
Source: PLoS Comput Biol. 2025 Oct 6;21(10):e1013451. doi: 10.1371/journal.pcbi.1013451 (PMC12513652; doi:10.1371/journal.pcbi.1013451)

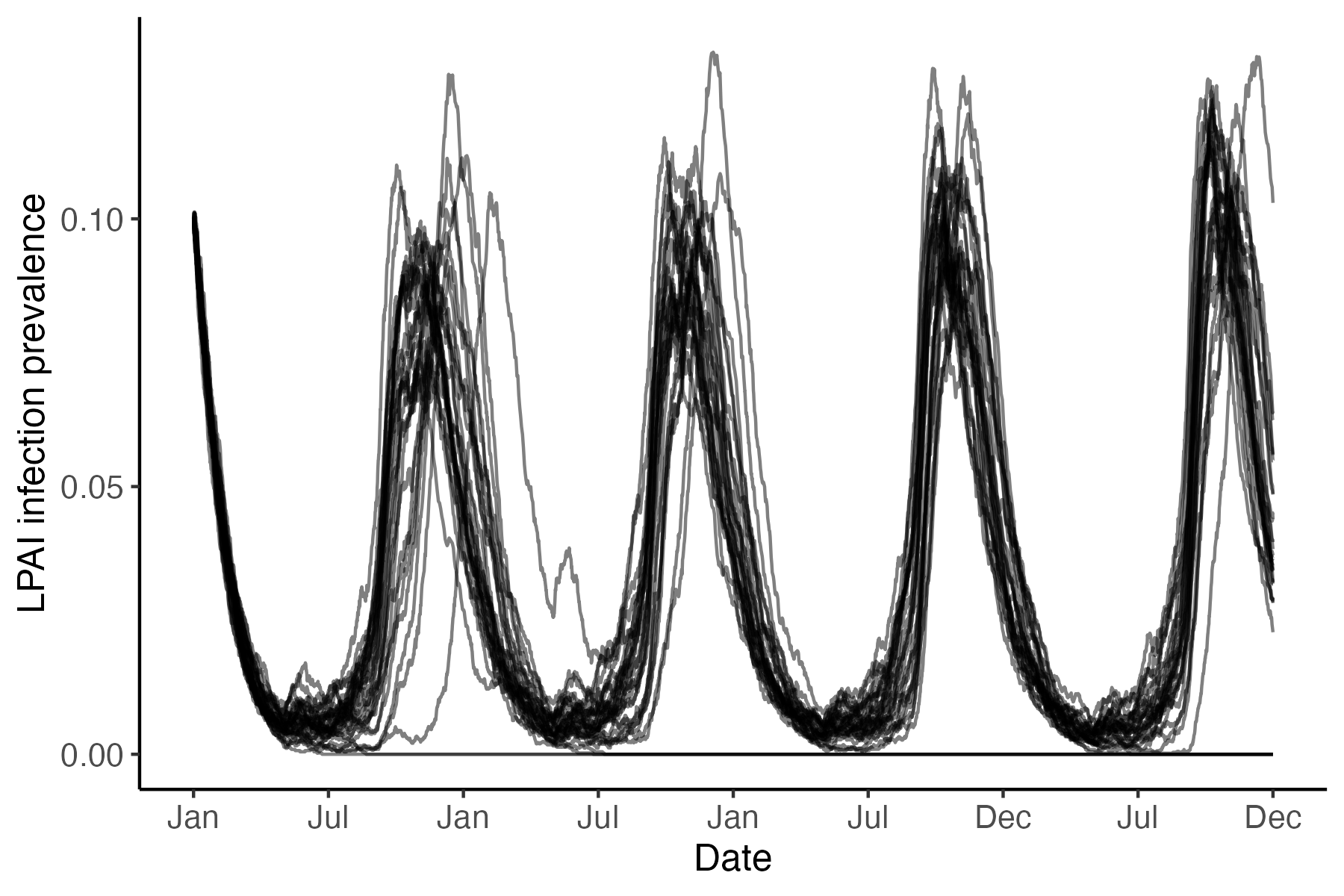

Supplement: S2 Fig — Each line represents a stochastic realization from the same parameter set. Parameters are shown in S1 Table. (PNG) [file pcbi.1013451.s003.png]

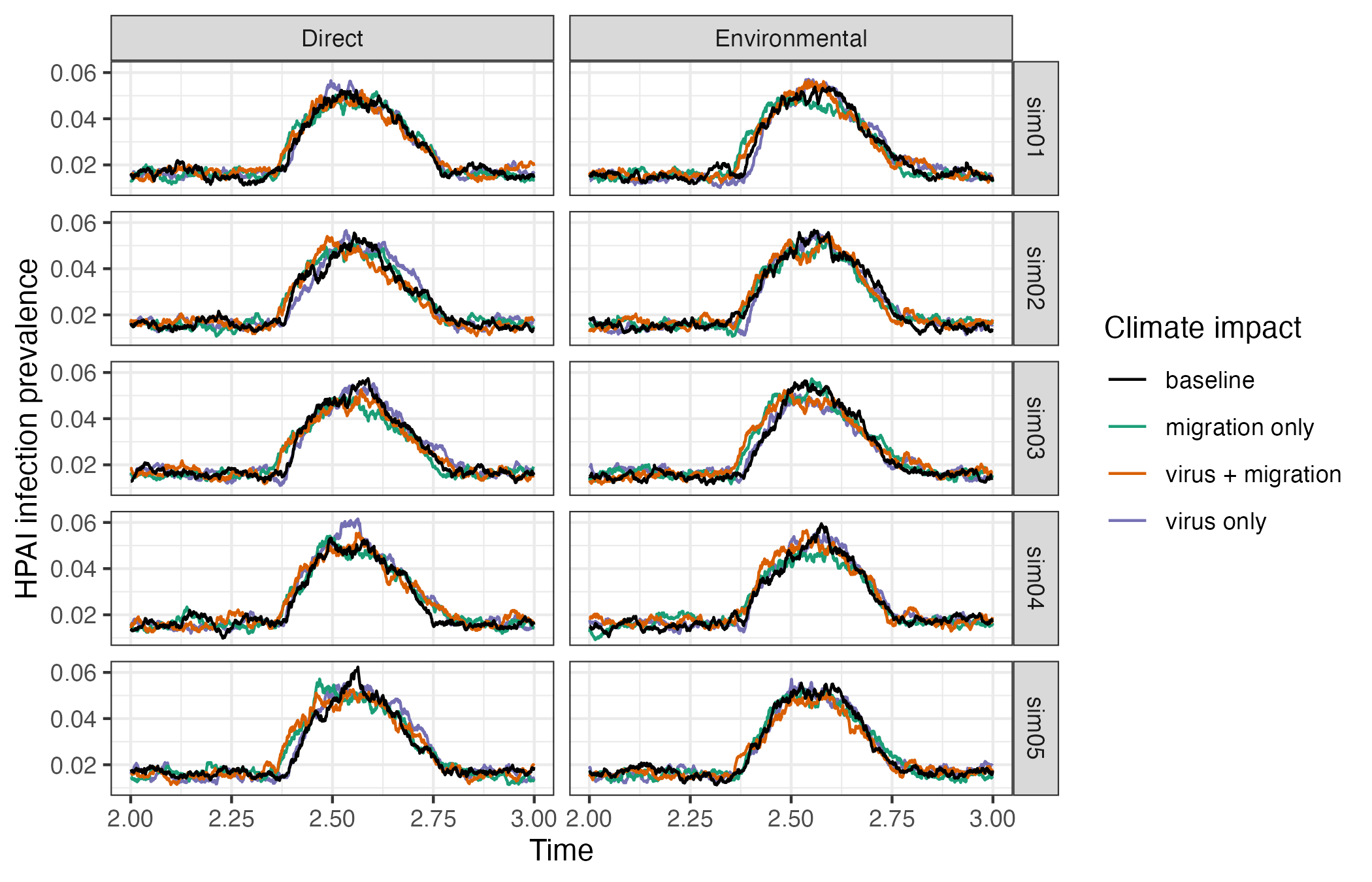

Supplement: S3 Fig — Each panel shows an example single simulation (of 20 runs for each HPAI strain). Columns show strains with different transmission modes (see Fig 6). (PNG) [file pcbi.1013451.s004.png]

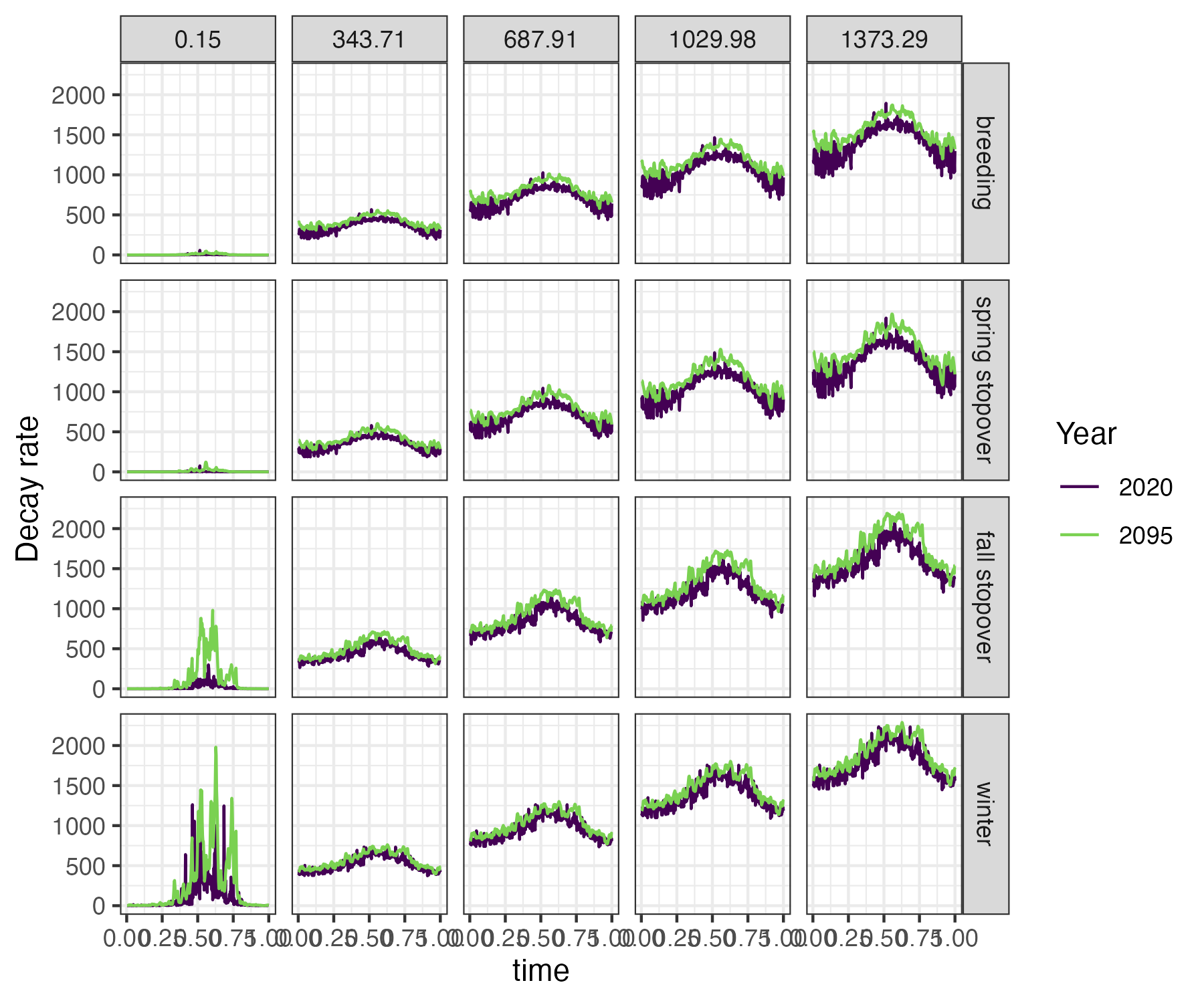

Supplement: S4 Fig — The y-axis shows the rate of viral decay in the environment. Columns show values of η2 and rows show sites. (PNG) [file pcbi.1013451.s005.png]

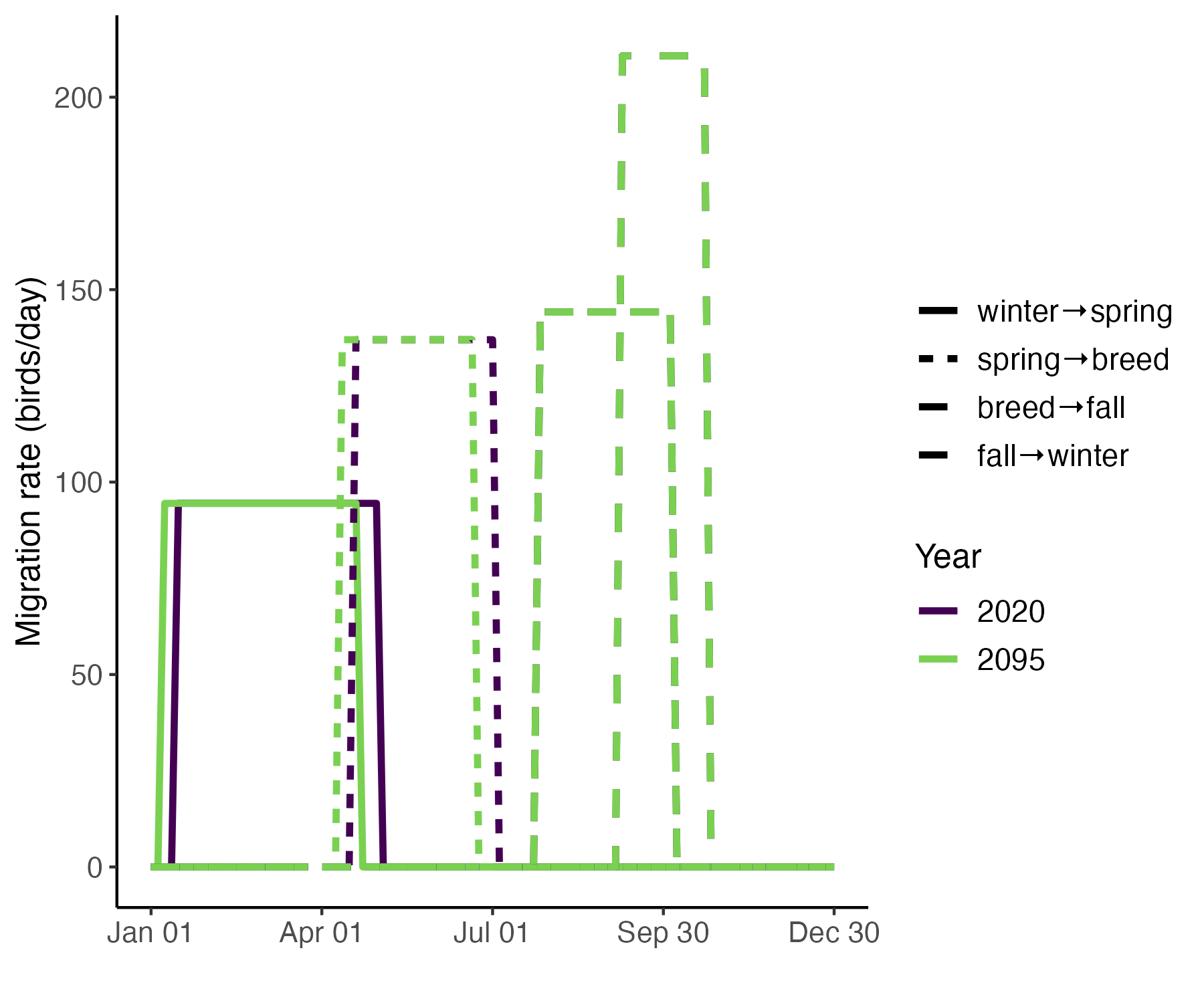

Supplement: S5 Fig — (PNG) [file pcbi.1013451.s006.png]

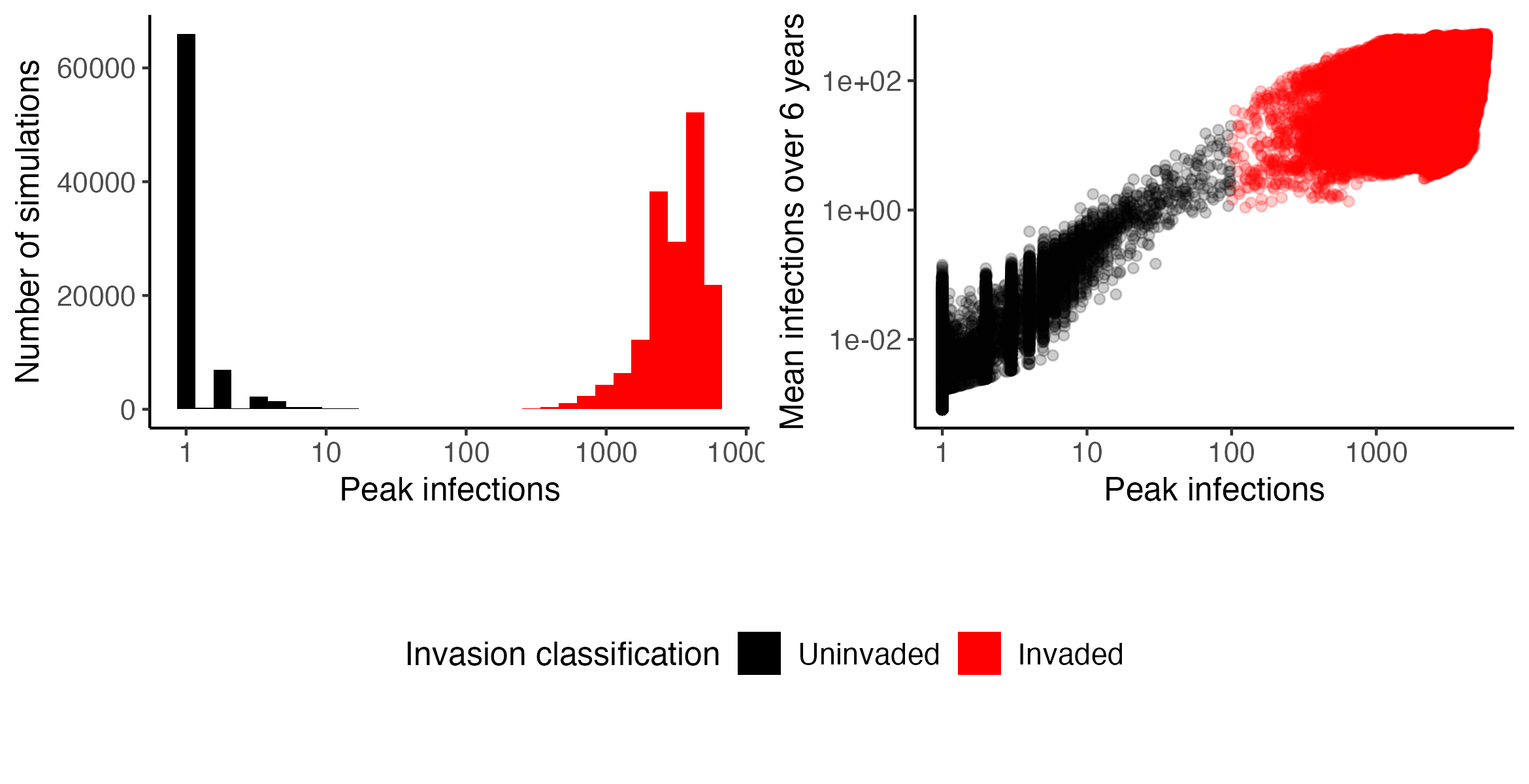

Supplement: S6 Fig — Strains with <100 peak infections were considered not to have successfully invaded the population (left). Strains with <100 initial infections had an average of <1 infection over the course of the six-year simulation (right). Note the log10 scale of peak and mean infections. (PNG) [file pcbi.1013451.s007.png]

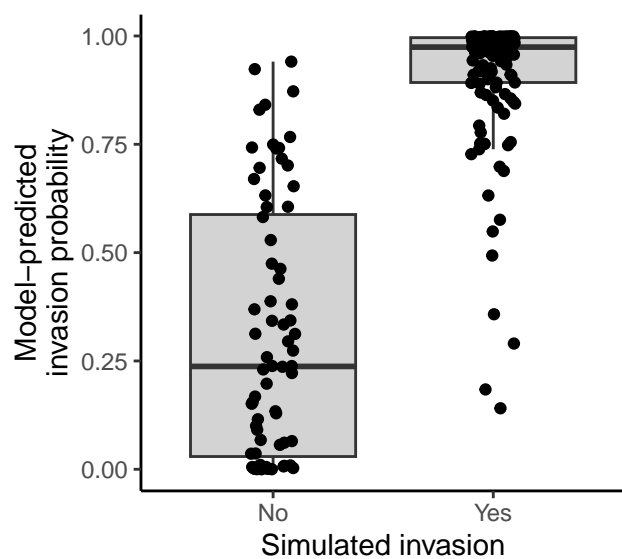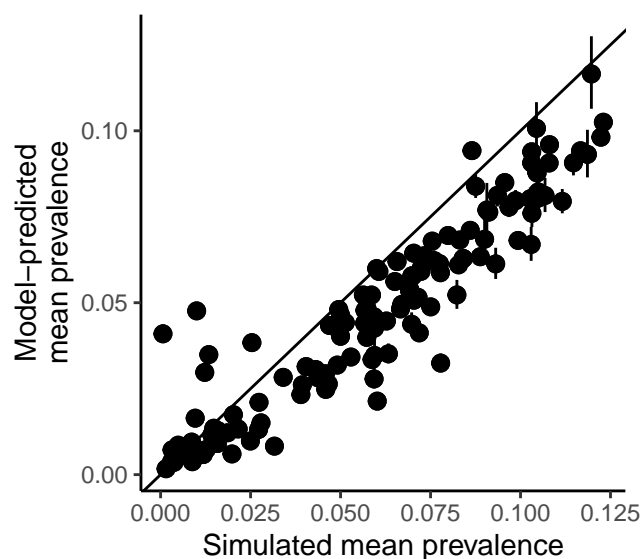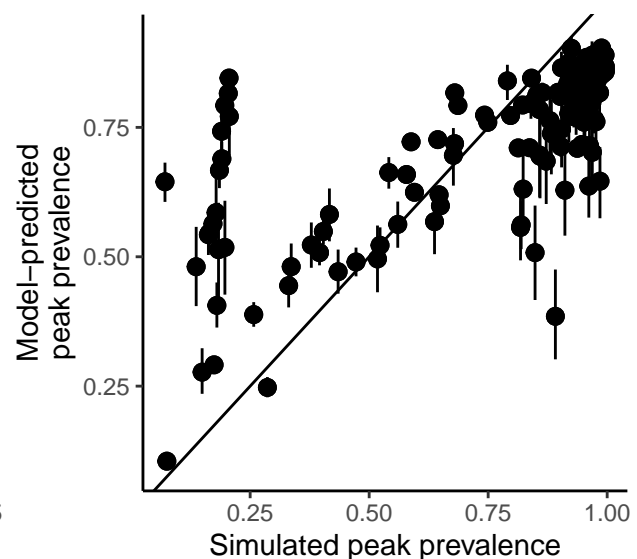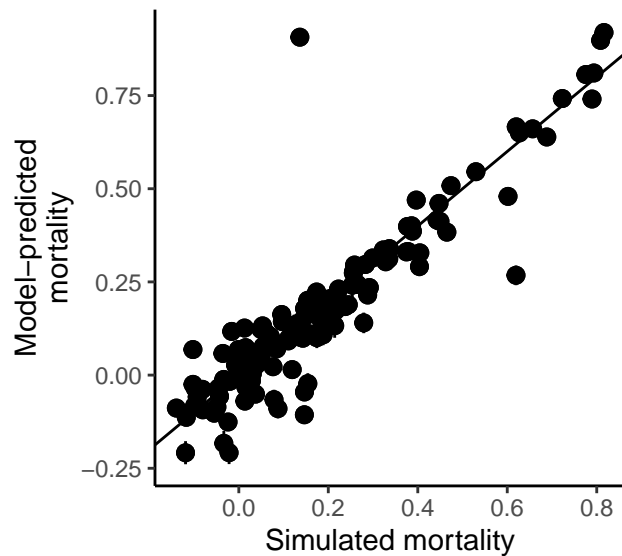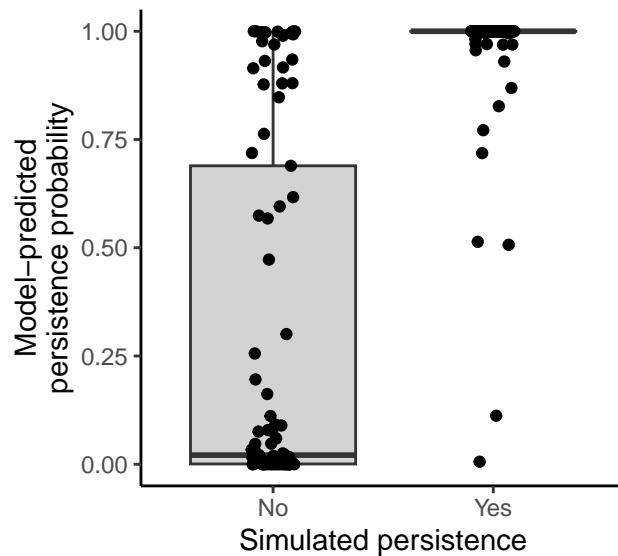

Supplement: S7 Fig — Model-predicted values show the expected values from generalized additive models, based on parameter values for each simulation. Error bars show 95% confidence intervals of the mean. Simulated values are actual outcomes from stochastic simulations. For continuous variables, the line shows a 1:1 relationship; for a perfect model, all points would fall on this line. (PDF) [file pcbi.1013451.s008.pdf]

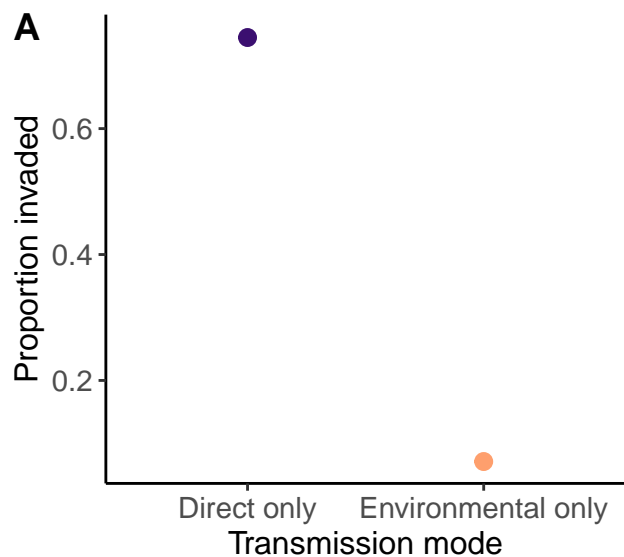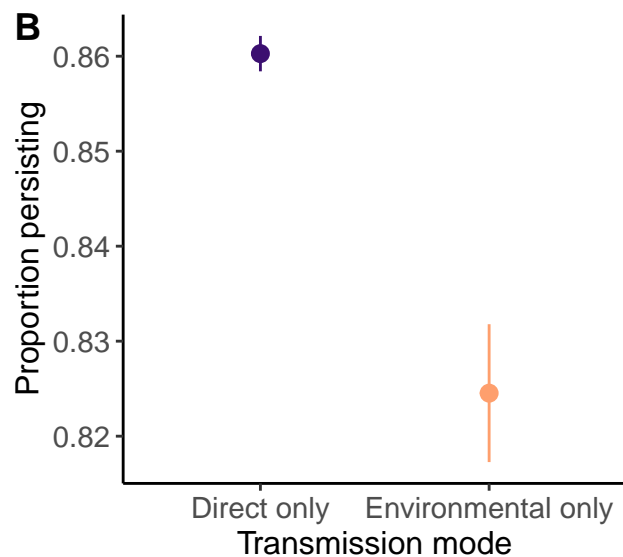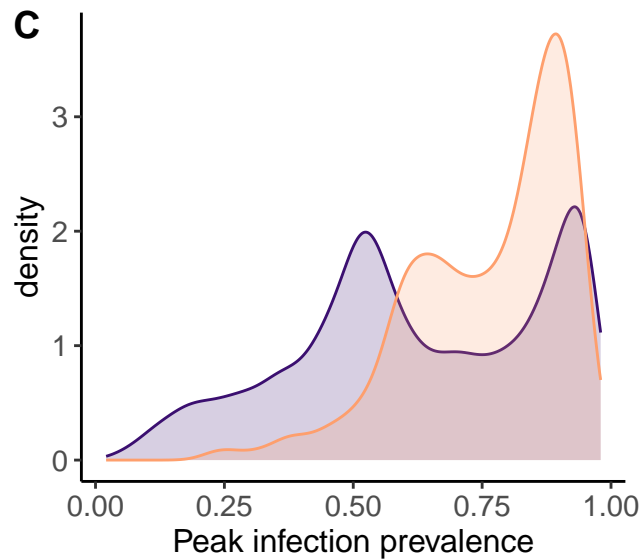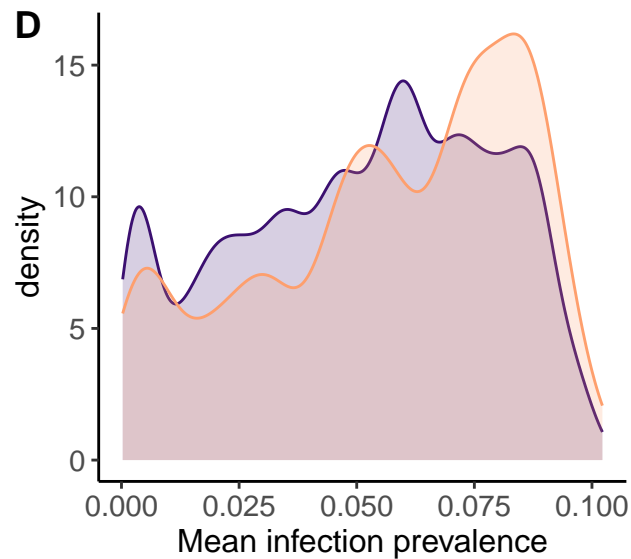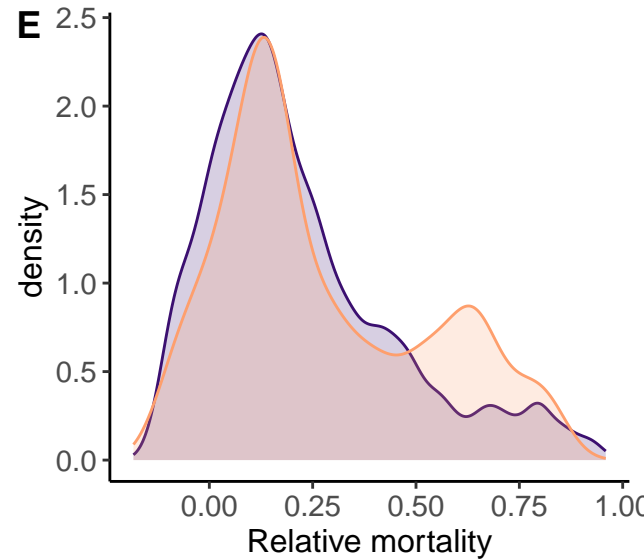

Supplement: S8 Fig — Points and error bars show means and 95% confidence intervals from raw data (error bars in (A) are too small to be visible). Density plots show distributions of raw data. All other parameters were randomly sampled from their distributions. (PDF) [file pcbi.1013451.s009.pdf]

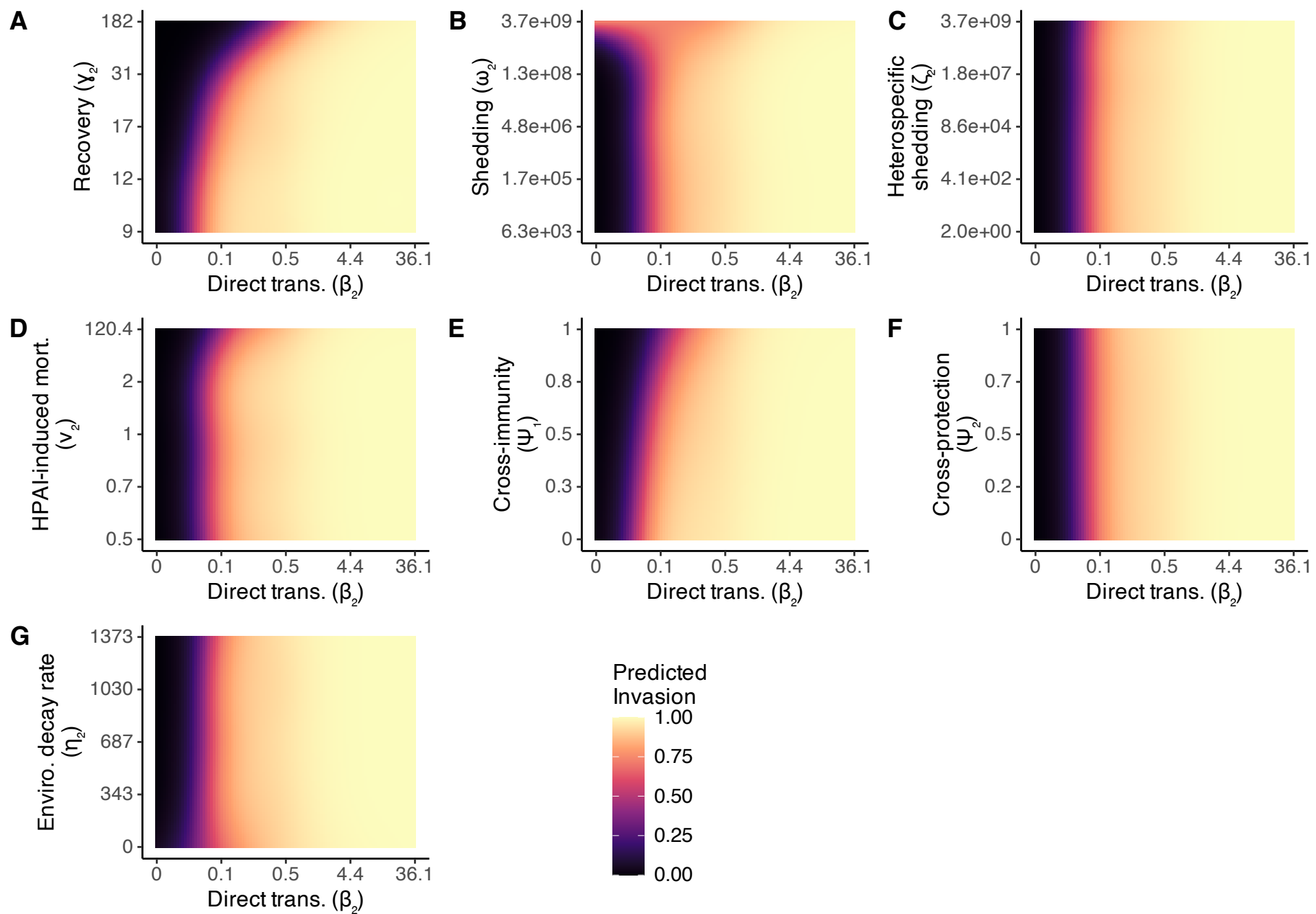

Supplement: S9 Fig — Colors within each plot show the fitted probability of invasion, as estimated from a generalized additive model, as a function of the direct transmission rate (x) and another HPAI trait (y). All traits not shown are held at their median values, except β2, which is held at 10-1.5. Plots show fitted values for a strain introduced at the breeding site on September 13. (PDF) [file pcbi.1013451.s010.pdf]

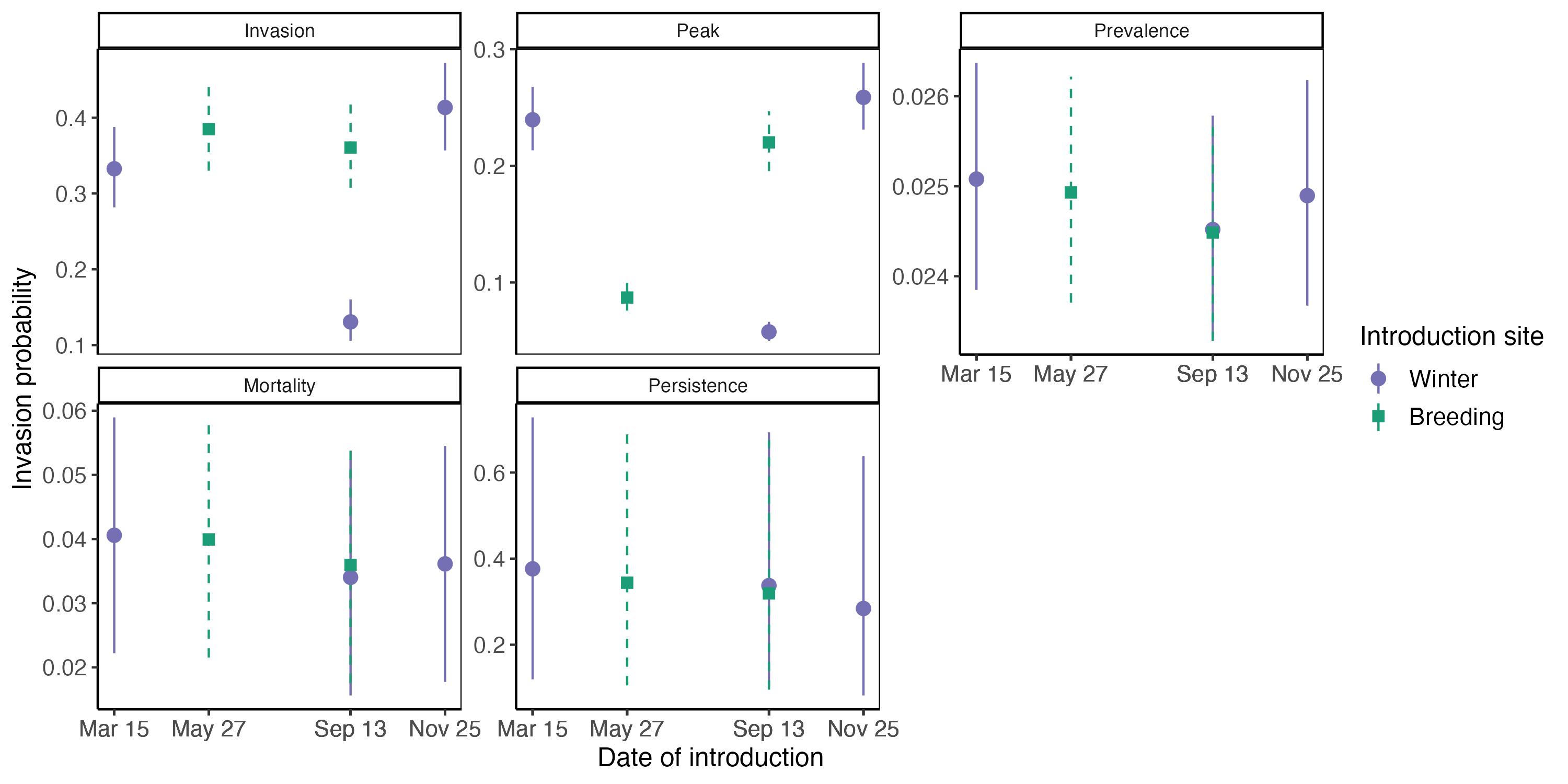

Supplement: S10 Fig — The y-axis shows the predictions from a generalized additive model, as a function of the combination of introduction date and location. Error bars shown 95% confidence intervals of the mean. All parameters not shown (i.e., HPAI traits) are held at their median values, except β2, which is held at 10-1.5. (PNG) [file pcbi.1013451.s011.png]

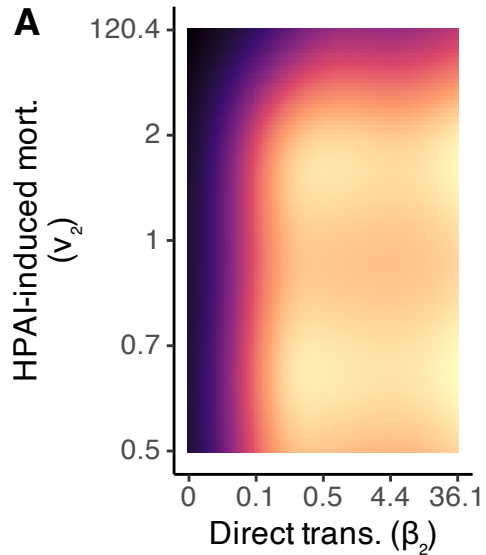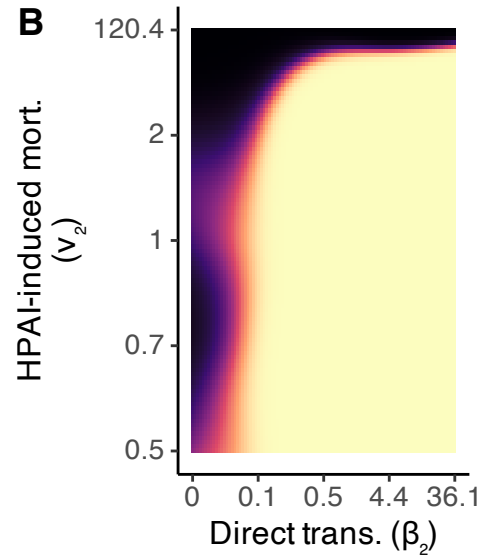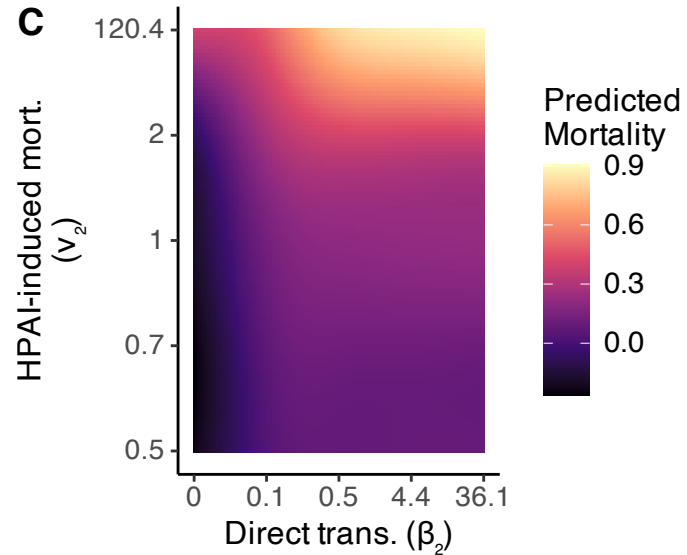

Supplement: S11 Fig — (A) Prevalence and (B) persistence are low for strains that cause high mortality, especially when direct transmission rates are high. (C) The combination of direct transmission and high HPAI-induced mortality rates increase mortality, sometimes to the point of population extinction (mortality = 1). Each plot shows predicted outcomes from a generalized additive model that evaluated persistence duration as a function of viral traits and time/location of introduction, including pairwise interactions. All continuous parameters not shown are held at their median values, except β2, which is held at 10-1.5. Plots show fitted values for a strain introduced at the breeding site on September 13 (at the end of the breeding season). (PDF) [file pcbi.1013451.s012.pdf]

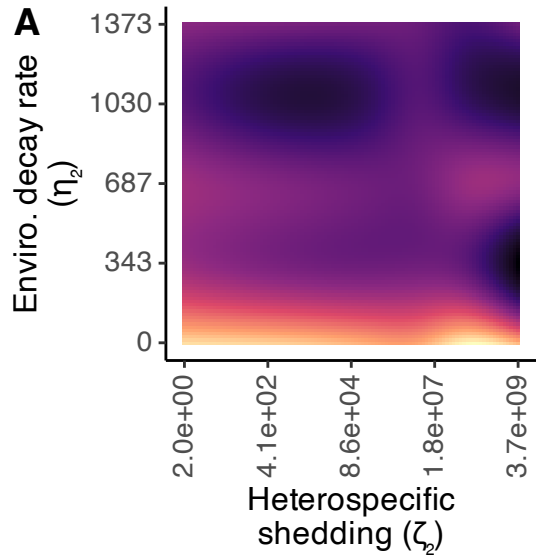

Predicted  
Prevalence

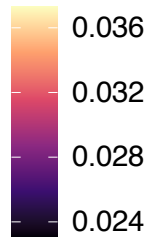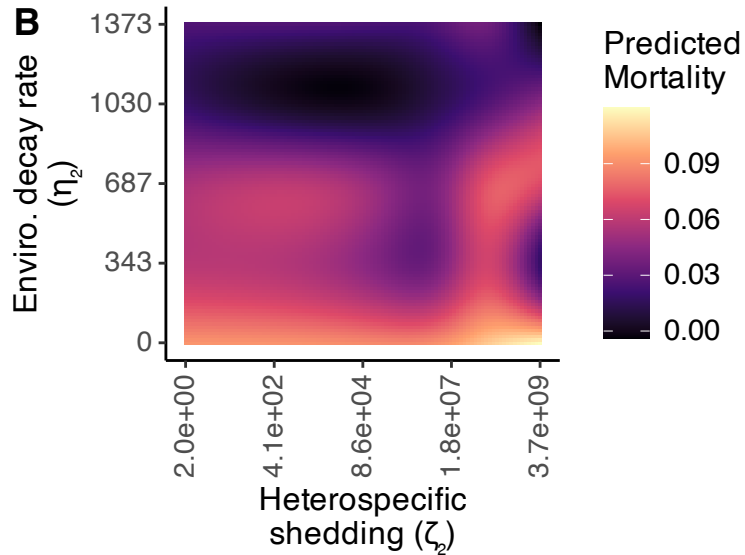

Predicted  
Mortality

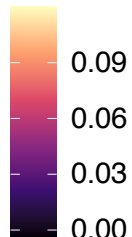

Supplement: S12 Fig — Note the low variation in values (i.e., range of color scale) in (A). Each plot shows predicted outcomes from a generalized additive model that modeled each outcome as a function of viral traits and time/location of introduction, including pairwise interactions. All continuous parameters not shown are held at their median values, except β2, which is held at 10-1.5. Plots show fitted values for a strain introduced at the breeding site on September 13 (at the end of the breeding season). (PDF) [file pcbi.1013451.s013.pdf]

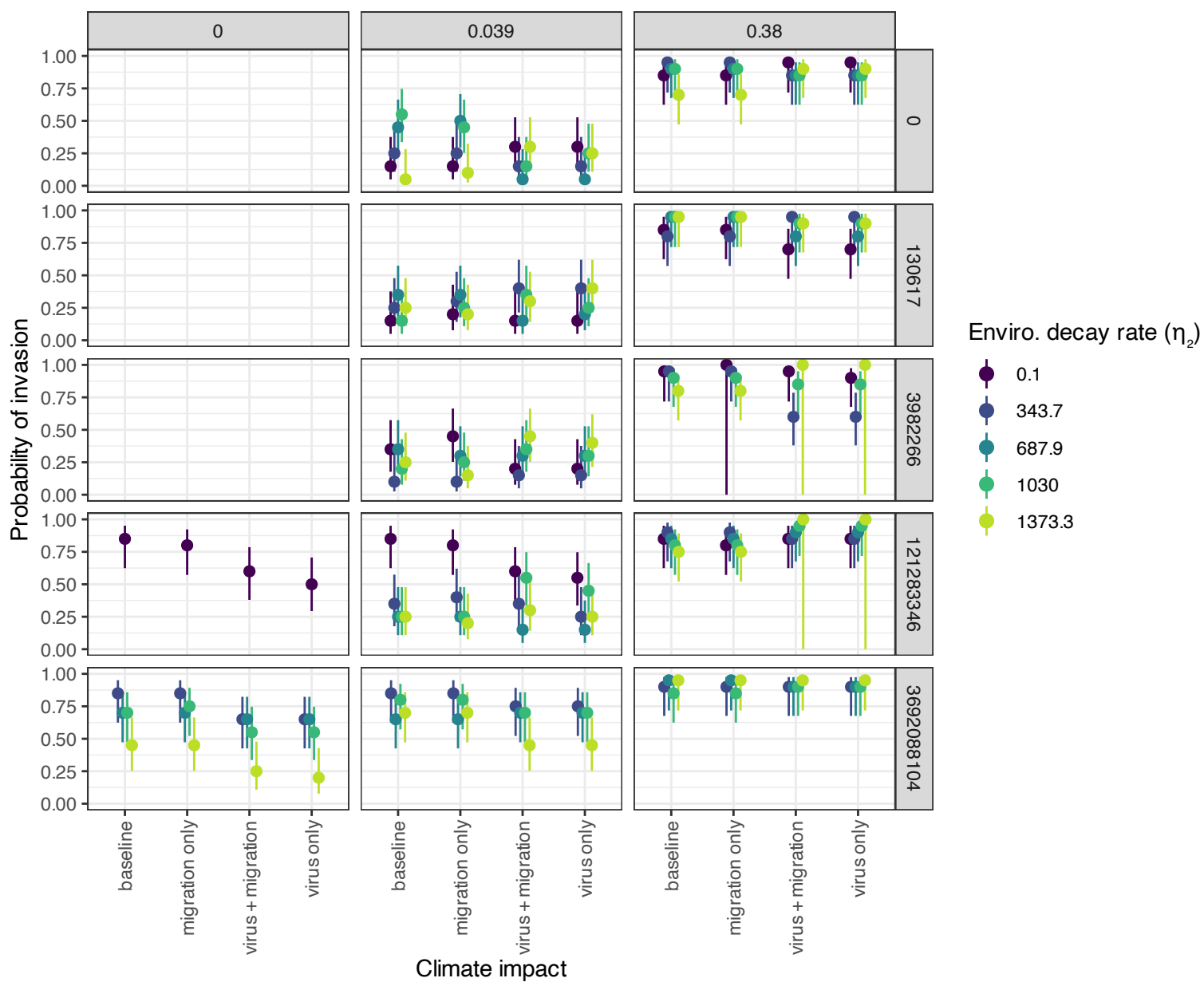

Supplement: S13 Fig — Results are from a generalized linear model that modeled invasion as a function of strain and its interaction with climate impact (x-axis). Columns show direct transmission rates (values of β2); only the three lowest values are shown because higher values of β2 resulted in 100% invasion. Missing panels or points indicate strains without sufficient data to fit a model (e.g., very low invasion probability).Rows show shedding rates (ω2). Colors show environmental decay rates, which are inversely related to temperature sensitivity (i.e., strains with low decay rates are the most temperature sensitive). (PDF) [file pcbi.1013451.s014.pdf]

Change in peak infection prevalence (year 0)

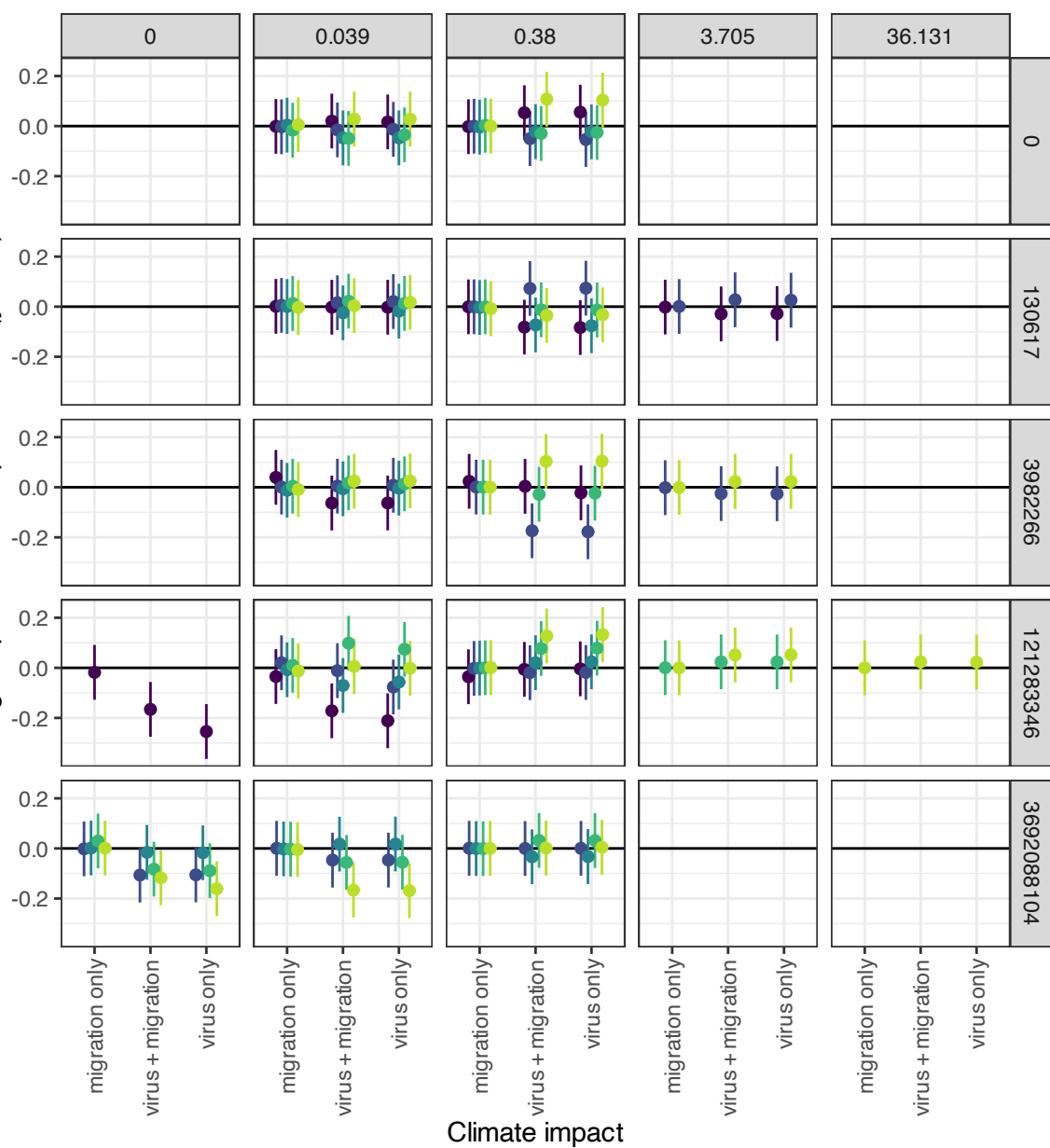

Enviro. decay rate ( $\eta_2$ )

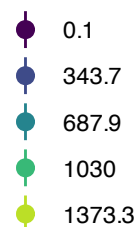

Supplement: S14 Fig — The y-axis shows the expected change in peak infection prevalence between 2020 and 2095. Results are from a generalized linear model that modeled invasion as a function of strain and its interaction with climate impact (x-axis). Columns show direct transmission rates (values of β2); rows show shedding rates (ω2). Colors show environmental decay rates, which are inversely related to temperature sensitivity (i.e., strains with low decay rates are the most temperature sensitive). Missing panels or points indicate strains without sufficient data to fit a model (e.g., low invasion probability). Stars indicate the two strains shown in the main text. (PDF) [file pcbi.1013451.s015.pdf]

Change in outbreak size (prevalence, year 2)

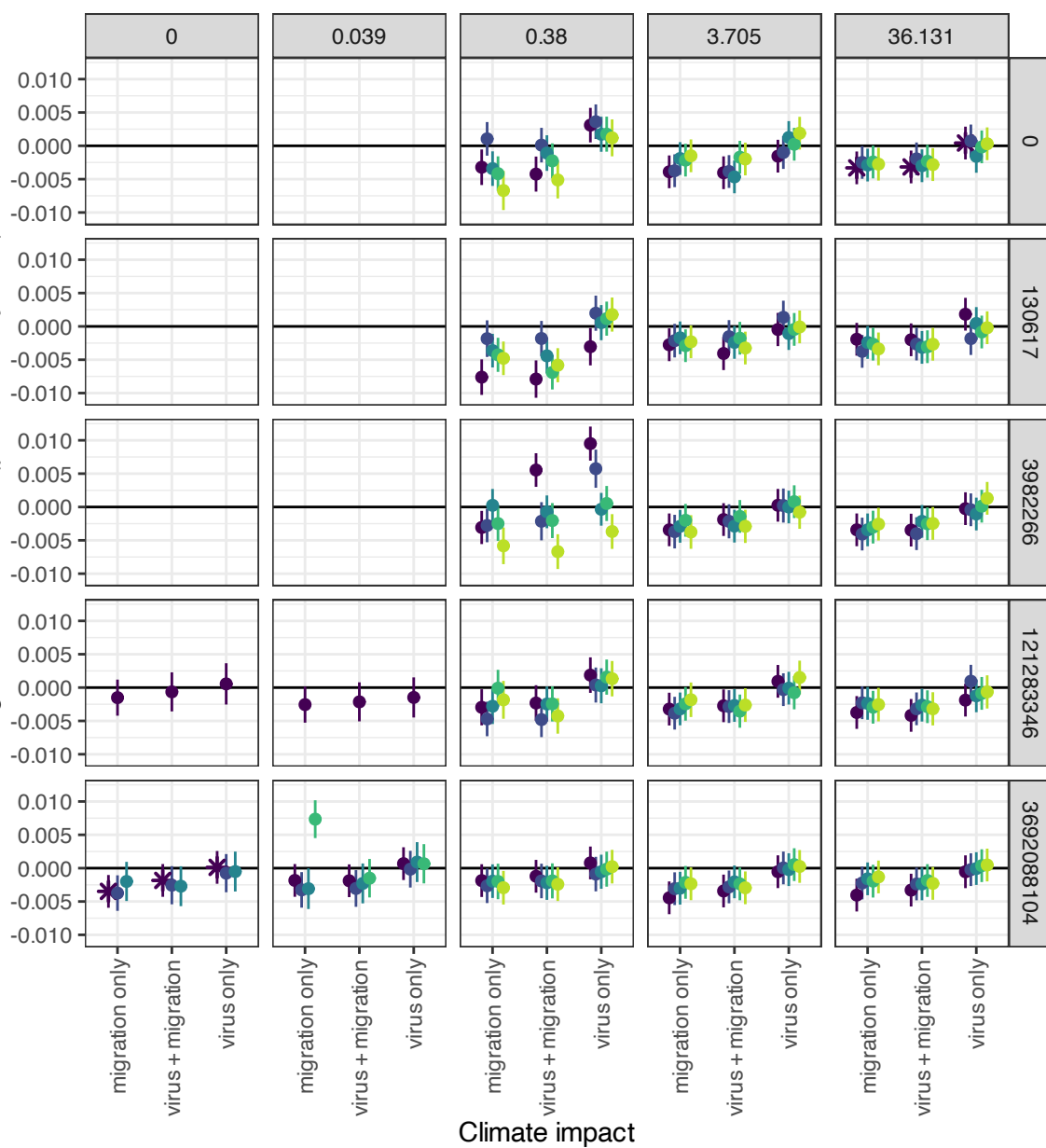

Supplement: S15 Fig — The y-axis shows the expected change in outbreak size between 2020 and 2095. Results are from a linear model that modeled outbreak size as a function of strain and its interaction with climate impact (x-axis). Columns show direct transmission rates (values of β2); rows show shedding rates (ω2). Colors show environmental decay rates, which are inversely related to temperature sensitivity (i.e., strains with low decay rates are the most temperature sensitive). Missing panels or points indicate strains without sufficient data to fit a model (e.g., low invasion probability). Stars indicate the two strains shown in the main text. (PDF) [file pcbi.1013451.s016.pdf]

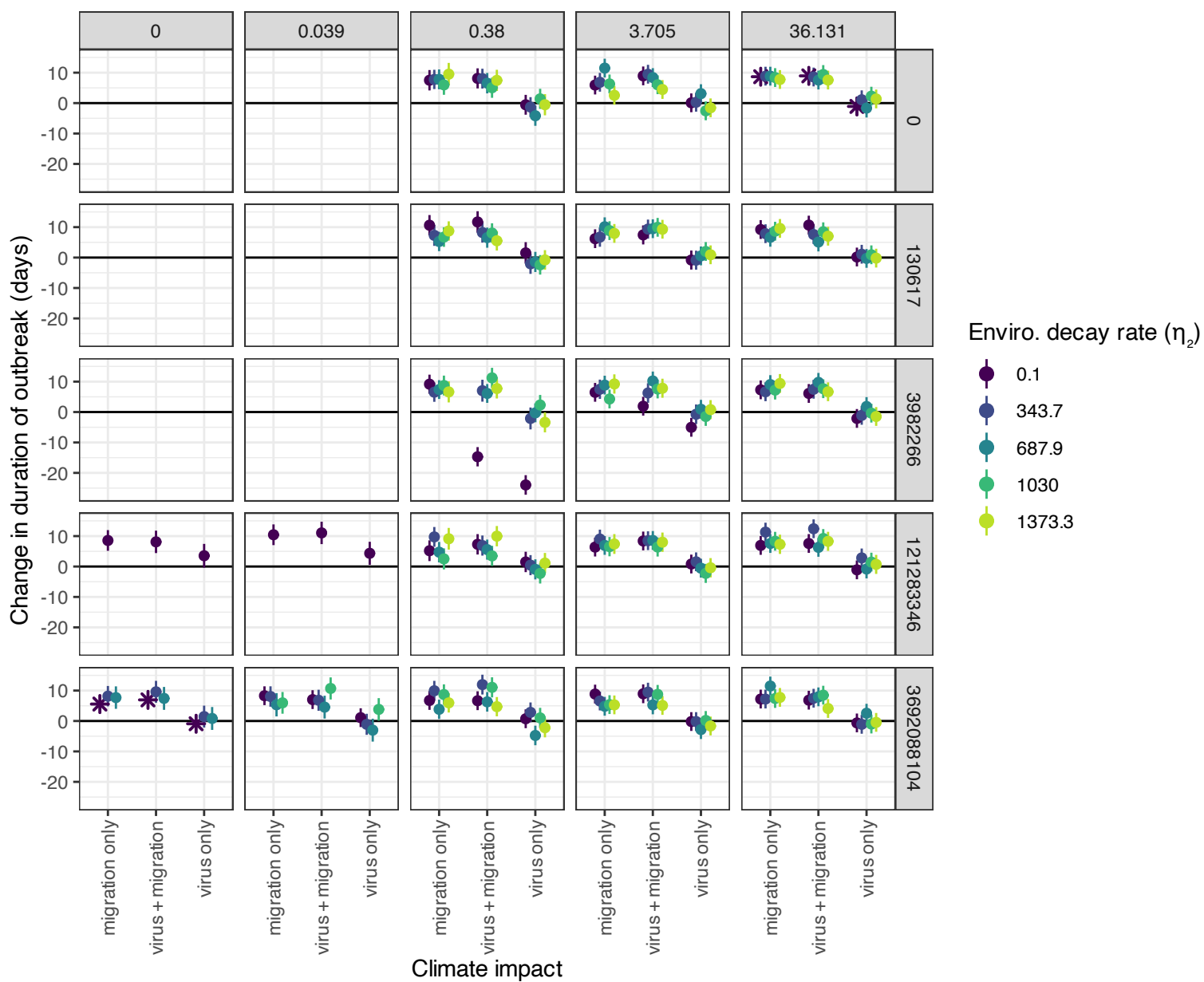

Supplement: S16 Fig — The y-axis shows the expected change in outbreak duration between 2020 and 2095. Results are from a linear model that modeled outbreak duration as a function of strain and its interaction with climate impact (x-axis). Columns show direct transmission rates (values of β2); rows show shedding rates (ω2). Colors show environmental decay rates, which are inversely related to temperature sensitivity (i.e., strains with low decay rates are the most temperature sensitive). Missing panels or points indicate strains without sufficient data to fit a model (e.g., low invasion probability). Stars indicate the two strains shown in the main text. (PDF) [file pcbi.1013451.s017.pdf]

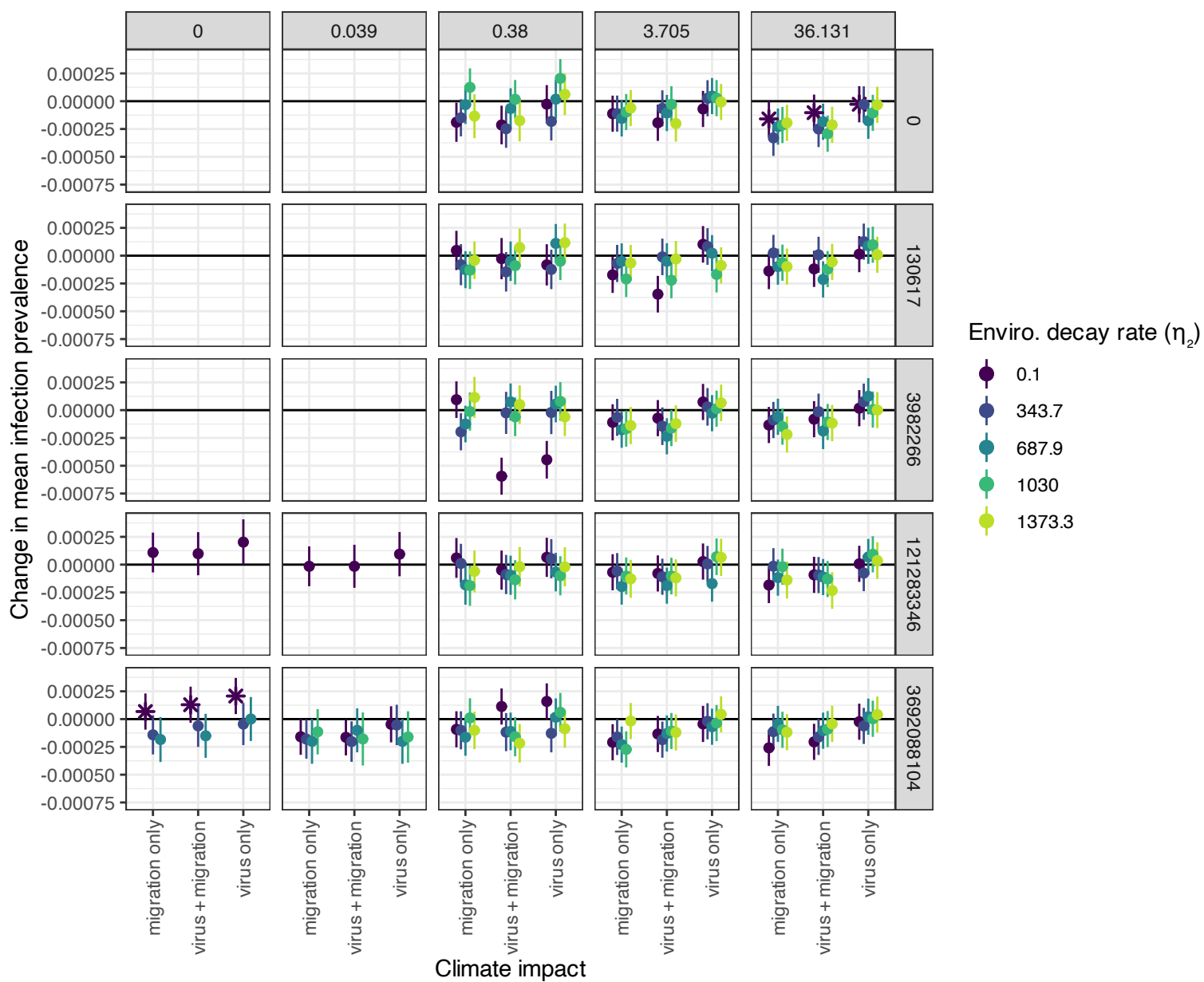

Supplement: S17 Fig — The y-axis shows the expected change in infection prevalence between 2020 and 2095. Results are from a linear model that modeled infection prevalence as a function of strain and its interaction with climate impact (x-axis). Columns show direct transmission rates (values of β2); rows show shedding rates (ω2). Colors show environmental decay rates, which are inversely related to temperature sensitivity (i.e., strains with low decay rates are the most temperature sensitive). Missing panels or points indicate strains without sufficient data to fit a model (e.g., low invasion probability). Stars indicate the two strains shown in the main text. (PDF) [file pcbi.1013451.s018.pdf]

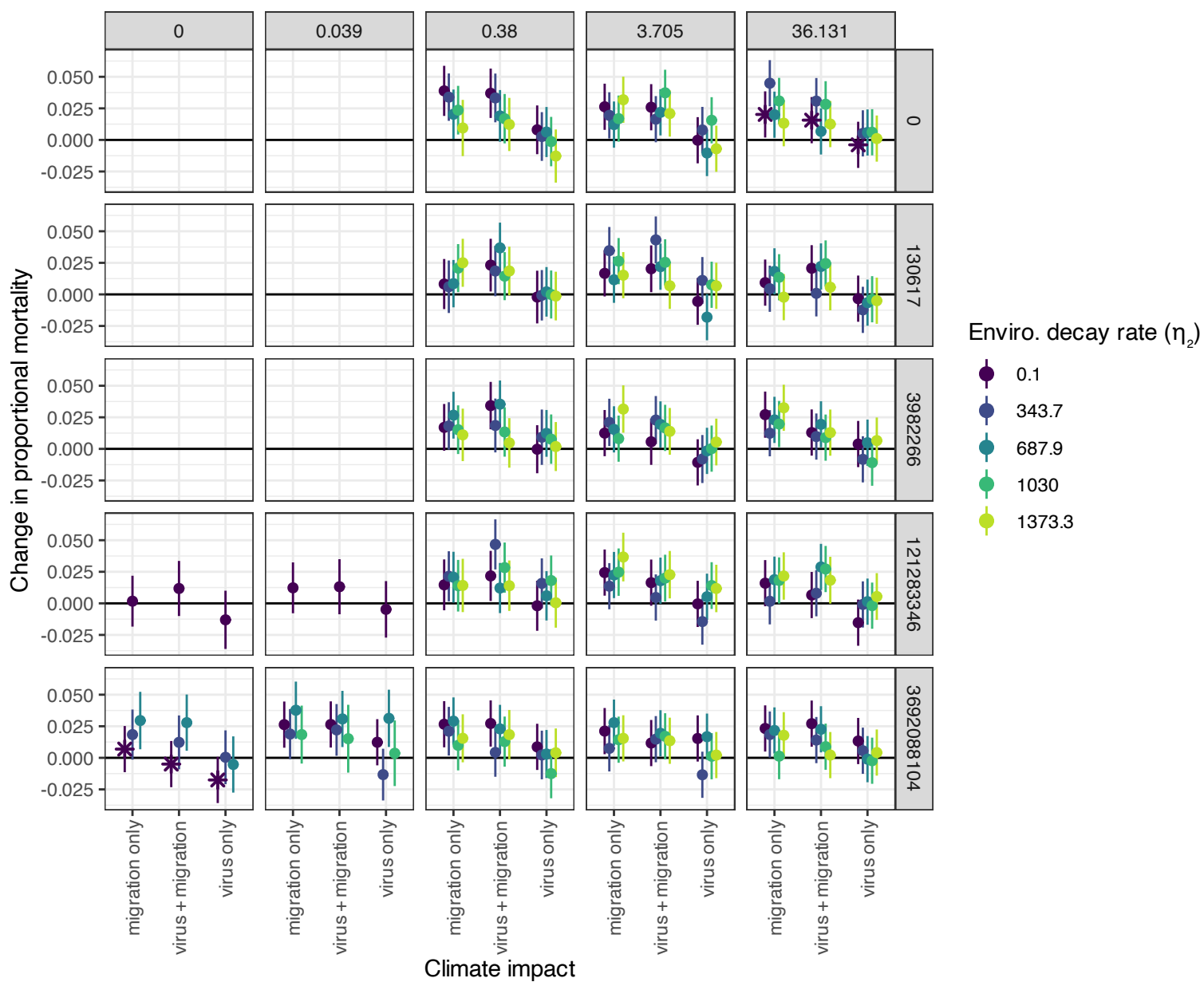

Supplement: S18 Fig — The y-axis shows the expected change in mortality between 2020 and 2095. Results are from a linear model that mortality as a function of strain and its interaction with climate impact (x-axis). Columns show direct transmission rates (values of β2); rows show shedding rates (ω2). Colors show environmental decay rates, which are inversely related to temperature sensitivity (i.e., strains with low decay rates are the most temperature sensitive). Missing panels or points indicate strains without sufficient data to fit a model (e.g., low invasion probability). Stars indicate the two strains shown in the main text. (PDF) [file pcbi.1013451.s019.pdf]
