# Supplementary material for: Host responses and viral traits interact to shape the impacts of climate warming on highly pathogenic avian influenza in migratory waterfowl
Source: PLoS Comput Biol. 2025 Oct 6;21(10):e1013451. doi: 10.1371/journal.pcbi.1013451 (PMC12513652; doi:10.1371/journal.pcbi.1013451)
Supplement: S1 Table — “Scale” gives the transformation used for parameter ranges and statistical modeling (e.g., β2 was varied between 10-4 and 4.7 on a log10 scale). “Value used in HPAI models” defines the baseline LPAI strain. See Gonnerman et al. [50] for a full review of challenge studies. (DOCX) [file pcbi.1013451.s020.docx]

**Host responses and viral traits interact to shape the impacts of climate warming on highly pathogenic avian influenza in migratory waterfowl**

Claire S. Teitelbaum, Michael L. Casazza, Cory T. Overton, Elliott L. Matchett, Diann J. Prosser

**S1 Table:** Parameters used in baseline simulations of low pathogenic and highly pathogenic avian influenza. “Scale” gives the transformation used for parameter ranges and statistical modeling (e.g., $\beta_{2}$ was varied between 10^-4^ and 4.7 on a log_10_ scale). “Value used in HPAI models” defines the baseline LPAI strain. See Gonnerman *et al.* [1] for a full review of challenge studies.

| **Parameter** | **Definition** | **Units** | **Scale** | **Minimum value** | **Min. reference** | **Maximum value** | **Max. reference** | **Additional references** | **Value used in HPAI models** |
| --- | --- | --- | --- | --- | --- | --- | --- | --- | --- |
| $N_{0}$ | starting population size | bird | Identity | 5000 | 5000 |  |  | [2,3] | 5000 |
| $\mu$ | host mortality rate (1/lifespan) | year^-1^ | Inverse | 0.25 | [4] | 0.34 | [4] |  | 0.31 |
| $\rho$ | consumption rate | year^-1^ | log_10_ | 1.2*10^-6^ | [5] | 1.2*10^-6^ | [6] | [7] | 1.2*10^-6^ |
| $\beta_{1}$ | LPAI contact/direct transmission rate | bird^-1^year^-1^ | log_10_ | 0 (no direct transmission) | [8] | 10 | [9] |  | 3.6*10^-2^ |
| $\beta_{2}$ | HPAI contact/direct transmission rate | bird^-1^year^-1^ | log_10_ | 0 (no direct transmission) | [8] | 36 | Parameterized relative to LPAI |  |  |
| $\gamma_{1}$ | recovery rate from LPAI infection (1/infectious period) | year^-1^ | inverse | 17 | [10] | 120 | [11,12] | [13] | 17.65 |
| $\gamma_{2}$ | recovery rate from HPAI infection (1/infectious period) | year^-1^ | inverse | 8.53 | Parameterization; [14] | 182.27 | Parameterization; [15] | [13,16–21] |  |
| $\kappa_{1}$ | LPAI infectious dose (BID_50_), determines shape of environmental infectiousness | ID_50_ | log_10_ | 100 | [22,23] | 100 | [22,24] |  | 100 |
| $\kappa_{2}$ | HPAI infectious dose (BID_50_), determines shape of environmental infectiousness | ID_50_ | log_10_ | 100 | Same as for LPAI | 100 | Same as for LPAI |  | 100 |
| $\epsilon_{1}$ | rate of waning LPAI immunity (1/immune duration) | year^-1^ | inverse | 0.5 | [11] | 13 | [25] | [26] | 0.55 |
| $\epsilon_{2}$ | rate of waning HPAI immunity | year^-1^ | inverse | 0.55 | Same as for LPAI | 0.55 | Same as for LPAI |  | 1.3 |
| $\omega_{1}$ | *per capita* LPAI viral shedding rate | ID_50_ year^-1^ | log_10_ | 0 (no environmental transmission) | [8] | 10^10^ | [23,27] |  | 3.7*10^4^ |
| $\omega_{2}$ | *per capita* HPAI viral shedding rate | ID_50_ year^-1^ | log_10_ | 0 (no environmental transmission) | Parameterized relative to LPAI; [28] | 3.7*10^9^ | Parameterized relative to LPAI |  |  |
| $\eta_{1}$ (intercept) | temperature-dependent LPAI decay rate in environment at 0°C | ID_50_ year^-1^ | log_10_ | 0.07 | [29] | 36.46 | [29] |  | 13.73 |
| $\eta_{2}$ (intercept) | temperature-dependent HPAI decay rate in environment at 0°C | ID_50_ year^-1^ | log_10_ | 0.13 | Parameterized relative to LPAI | 1373 | Parameterized relative to LPAI |  |  |
| $\zeta_{1}$ | heterospecific shedding rate of LPAI | ID_50_ year^-1^ | log_10_ | 0 (no shedding) |  | 10^4^ | Parameterized relative to $\omega_{1}$ |  | 680 |
| $\zeta_{2}$ | heterospecific shedding rate of HPAI | ID_50_ year^-1^ | log_10_ | 0 (no shedding) | Parameterized relative to LPAI | 6.8*10^4^ | Parameterized relative to LPAI |  |  |
| $\psi_{1}$ | strength of cross-protection from infection | none | identity | 0 | Full range of possible values | 1 | Full range of possible values |  |  |
| $\psi_{2}$ | strength of cross-protection from mortality | none | identity | 0 | Full range of possible values | 1 | Full range of possible values | [15] |  |
| $\nu_{2}$ | host mortality rate from HPAI infection (1/survival duration) | year^-1^ | inverse | 0.5 | [22,30–32] | 121 | [10,15] | [19,20,33] |  |

1. Gonnerman M, Leyson C, Sullivan JD, Pantin-Jackwood MJ, Spackman E, Mullinax JM, et al. A systematic review of laboratory investigations into the pathogenesis of avian influenza viruses in wild avifauna of North America. Proceedings of the Royal Society B: Biological Sciences. 2024;291: 20241845. doi:10.1098/rspb.2024.1845

2. Pacific Flyway Council. Pacific Flyway management plan for the greater white-fronted goose. Portland, OR: Pacific Flyway Council, U.S. Fish and Wildlife Service, Canadian Wildlife Service, Dirección General de Conservación Ecológica de Recursos Naturales; 2003 July p. 27.

3. Ely CR, Dzubin AX, Mlodinow SG, Kirwan GM, Carboneras C, Garcia E. Greater White-fronted Goose (Anser albifrons). In: Billerman SM, Keeney BK, Rodewald PG, Schulenberg TS, editors. Birds of the World. Cornell Lab of Ornithology; 2024. doi:10.2173/bow.gwfgoo.01.1

4. Ely CR, Dzubin AX, Carboneras C, Kirwan GM, Garcia E. Greater White-fronted Goose (Anser albifrons). In: Billerman SM, Keeney BK, Rodewald PG, Schulenberg TS, editors. Birds of the World. Cornell Lab of Ornithology; 2020. doi:10.2173/bow.gwfgoo.01

5. Brown VL, Drake JM, Barton HD, Stallknecht DE, Brown JD, Rohani P. Neutrality, cross-immunity and subtype dominance in avian influenza viruses. PLoS ONE. 2014;9. doi:10.1371/journal.pone.0088817

6. Breban R, Drake JM, Stallknecht DE, Rohani P. The Role of Environmental Transmission in Recurrent Avian Influenza Epidemics. PLoS Computational Biology. 2009;5: e1000346. doi:10.1371/journal.pcbi.1000346

7. Roche B, Rohani P. Environmental transmission scrambles coexistence patterns of avian influenza viruses. Epidemics. 2010;2: 92–98. doi:10.1016/j.epidem.2010.03.002

8. Roche B, Lebarbenchon C, Gauthier-Clerc M, Chang CM, Thomas F, Renaud F, et al. Water-borne transmission drives avian influenza dynamics in wild birds: The case of the 2005-2006 epidemics in the Camargue area. Infection, Genetics and Evolution. 2009;9: 800–805. doi:10.1016/j.meegid.2009.04.009

9. Yin S, de Knegt HJ, de Jong MCM, Si Y, Prins HHT, Huang ZYX, et al. Effects of migration network configuration and migration synchrony on infection prevalence in geese. Journal of Theoretical Biology. 2020;502: 110315. doi:10.1016/j.jtbi.2020.110315

10. Bourouiba L, Teslya A, Wu J. Highly pathogenic avian influenza outbreak mitigated by seasonal low pathogenic strains: Insights from dynamic modeling. Journal of Theoretical Biology. 2011;271: 181–201. doi:10.1016/j.jtbi.2010.11.013

11. Lisovski S, van Dijk JGB, Klinkenberg D, Nolet BA, Fouchier RAM, Klaassen M. The roles of migratory and resident birds in local avian influenza infection dynamics. Journal of Applied Ecology. 2018;55: 2963–2975. doi:10.1111/1365-2664.13154

12. Shriner SA, Root JJ, Ellis JW, Bentler KT, VanDalen KK, Gidlewski T, et al. Influenza A virus surveillance, infection and antibody persistence in snow geese (*Anser caerulescens*). Transboundary and Emerging Diseases. 2021;69: 742–752. doi:10.1111/tbed.14044

13. Berhane Y, Embury-Hyatt C, Leith M, Kehler H, Suderman M, Pasick J. Pre-exposing Canada geese (Branta canadensis) to a low-pathogenic H1N1 avian influenza virus protects them against H5N1 HPAI virus challenge. Journal of Wildlife Diseases. 2014;50: 84–97. doi:10.7589/2012-09-237

14. Pasick J, Berhane Y, Embury-Hyatt C, Copps J, Kehler H, Handel K, et al. Susceptibility of Canada Geese (Branta canadensis) to Highly Pathogenic Avian Influenza Virus (H5N1). Emerg Infect Dis. 2007;13: 1821–1827. doi:10.3201/eid1312.070502

15. Berhane Y, Leith M, Embury-Hyatt C, Neufeld J, Babiuk S, Hisanaga T, et al. Studying possible cross-protection of Canada geese preexposed to North American low pathogenicity avian influenza virus strains (H3N8, H4N6, and H5N2) against an H5N1 highly pathogenic avian influenza challenge. Avian Diseases. 2010;54: 548–554. doi:10.1637/8841-040309-Reg.1

16. Brown JD, Stallknecht DE, Swayne DE. Experimental Infection of Swans and Geese with Highly Pathogenic Avian Influenza Virus (H5N1) of Asian Lineage. Emerging Infectious Diseases. 2008;14: 136–142.

17. Webster RG, Guan Y, Peiris M, Walker D, Krauss S, Zhou NN, et al. Characterization of H5N1 Influenza Viruses That Continue To Circulate in Geese in Southeastern China. Journal of Virology. 2002;76: 118–126. doi:10.1128/jvi.76.1.118-126.2002

18. Leigh Perkins LE, Swayne DE. Pathogenicity of a Hong Kong-origin H5N1 highly pathogenic avian influenza virus for emus, geese, ducks, and pigeons. Avian Diseases. 2002;46: 53–63. doi:10.1637/0005-2086(2002)046%5B0053:POAHKO%5D2.0.CO;2

19. Takekawa JY, Prosser DJ, Newman SH, Muzaffar SB, Hill NJ, Yan B, et al. Victims and vectors: Highly pathogenic avian influenza H5N1 and the ecology of wild birds. Avian Biology Research. 2010;3: 51–73. doi:10.3184/175815510X12737339356701

20. Tian G, Zhang S, Li Y, Bu Z, Liu P, Zhou J, et al. Protective efficacy in chickens, geese and ducks of an H5N1-inactivated vaccine developed by reverse genetics. Virology. 2005;341: 153–162. doi:10.1016/j.virol.2005.07.011

21. Eggert D, Swayne DE. Single vaccination provides limited protection to ducks and geese against H5N1 high pathogenicity avian influenza virus. Avian Diseases. 2010;54: 1224–1229. doi:10.1637/9410-052810-Reg.1

22. Aldous EW, Seekings JM, McNally A, Nili H, Fuller CM, Irvine RM, et al. Infection dynamics of highly pathogenic avian influenza and virulent avian paramyxovirus type 1 viruses in chickens, turkeys and ducks. Avian Pathology. 2010;39: 265–273. doi:10.1080/03079457.2010.492825

23. Rohani P, Breban R, Stallknecht DE, Drake JM. Environmental transmission of low pathogenicity avian influenza viruses and its implications for pathogen invasion. Proceedings of the National Academy of Sciences of the United States of America. 2009;106: 10365–10369. doi:10.1073/pnas.0809026106

24. Segovia KM, França MS, Leyson CL, Kapczynski DR, Chrzastek K, Bahnson CS, et al. Heterosubtypic immunity increases infectious dose required to infect mallard ducks with influenza a virus. PLoS ONE. 2018;13: 1–12. doi:10.1371/journal.pone.0196394

25. Vaidya NK, Wahl LM. Avian influenza dynamics under periodic environmental conditions. SIAM Journal on Applied Mathematics. 2015;75: 443–467. doi:10.1137/140966642

26. Homme PJ, Easterday BC. Avian influenza virus infections. IV. Response of pheasants, ducks, and geese to influenza A-turkey-Wisconsin-1966 virus. Avian diseases. 1970;14: 285–290. doi:10.2307/1588473

27. Webster RG, Yakhno M, Hinshaw VS, Bean WJ, Copal Murti K. Intestinal influenza: Replication and characterization of influenza viruses in ducks. Virology. 1978;84: 268–278. doi:10.1016/0042-6822(78)90247-7

28. Hénaux V, Samuel MD. Avian influenza shedding patterns in waterfowl: Implications for surveillance, environmental transmission, and disease spread. Journal of Wildlife Diseases. 2011;47: 566–578. doi:10.7589/0090-3558-47.3.566

29. Handel A, Lebarbenchon C, Stallknecht D, Rohani P. Trade-offs between and within scales: Environmental persistence and within-host fitness of avian influenza viruses. Proceedings of the Royal Society B: Biological Sciences. 2014;281. doi:10.1098/rspb.2013.3051

30. Van Den Brand JMA, Verhagen JH, Veldhuis Kroeze EJB, Van De Bildt MWG, Bodewes R, Herfst S, et al. Wild ducks excrete highly pathogenic avian influenza virus H5N8 (2014-2015) without clinical or pathological evidence of disease. Emerging Microbes and Infections. 2018;7. doi:10.1038/s41426-018-0070-9

31. Pillai SPS, Pantin-Jackwood M, Suarez DL, Saif YM, Lee CW. Pathobiological characterization of low-pathogenicity H5 avian influenza viruses of diverse origins in chickens, ducks and turkeys. Archives of Virology. 2010;155: 1439–1451. doi:10.1007/s00705-010-0727-8

32. Pantin-Jackwood MJ, Swayne DE. Pathogenesis and pathobiology of avian influenza virus infection in birds. OIE Scientific and Technical Review. 2009;28: 113–136.

33. Kwon YK, Thomas C, Swayne DE. Variability in pathobiology of South Korean H5N1 high-pathogenicity avian influenza virus infection for 5 species of migratory waterfowl. Veterinary Pathology. 2010;47: 495–506. doi:10.1177/0300985809359602
