# Supplementary material for: Host responses and viral traits interact to shape the impacts of climate warming on highly pathogenic avian influenza in migratory waterfowl
Source: PLoS Comput Biol. 2025 Oct 6;21(10):e1013451. doi: 10.1371/journal.pcbi.1013451 (PMC12513652; doi:10.1371/journal.pcbi.1013451)
Supplement: S2 Table — (DOCX) [file pcbi.1013451.s021.docx]

**Host responses and viral traits interact to shape the impacts of climate warming on highly pathogenic avian influenza in migratory waterfowl**

Claire S. Teitelbaum, Michael L. Casazza, Cory T. Overton, Elliott L. Matchett, Diann J. Prosser

**S2 Table**: Dates and locations of HPAI introduction in simulations measuring sensitivity of HPAI dynamics to viral traits. *t* corresponds to time (proportion of year) in model equations (see Supplementary Methods).

| **Calendar date** | ***t*** | **Site** | **Description** |
| --- | --- | --- | --- |
| March 15 | 0.2 | wintering | End of wintering |
| May 27 | 0.4 | breeding | Beginning of breeding |
| September 13 | 0.7 | breeding | End of breeding |
| September 13 | 0.7 | wintering | Beginning of wintering |
| November 25 | 0.9 | wintering | Mid-winter |
