# Supplementary material for: Host responses and viral traits interact to shape the impacts of climate warming on highly pathogenic avian influenza in migratory waterfowl
Source: PLoS Comput Biol. 2025 Oct 6;21(10):e1013451. doi: 10.1371/journal.pcbi.1013451 (PMC12513652; doi:10.1371/journal.pcbi.1013451)
Supplement: S3 Table — All other parameters were held constant (see S1 Table for LPAI parameters). In all simulations, strains were introduced at the breeding grounds on May 27 (t = 0.4). The viral decay rate provided (η2) is the viral decay rate at 0°C and is inversely related to temperature sensitivity. Simulations used pariwise combinations of these three parameters, for a total of 120 strains; simulations were not run for strains where both β2=0 and ω2= 0. See S1 Table for parameter definitions. Other HPAI parameter values were fixed at: γ2=32.14, ν2=1.68, ψ1=0.48, ψ2=0.89, ζ2=2418. (DOCX) [file pcbi.1013451.s022.docx]

**Host responses and viral traits interact to shape the impacts of climate warming on highly pathogenic avian influenza in migratory waterfowl**

Claire S. Teitelbaum, Michael L. Casazza, Cory T. Overton, Elliott L. Matchett, Diann J. Prosser

**S3 Table**: Parameter values used in simulations of climate change. All other parameters were held constant (see S1 Table for LPAI parameters). In all simulations, strains were introduced at the breeding grounds on May 27 (*t=*0.4). The viral decay rate provided ($\eta_{2}$) is the viral decay rate at 0°C and is inversely related to temperature sensitivity. Simulations used pariwise combinations of these three parameters, for a total of 120 strains; simulations were not run for strains where both $\beta_{2}=0$ and $\omega_{2}$= 0. See Table S1 for parameter definitions. Other HPAI parameter values were fixed at: $\gamma_{2}=32.14, \nu_{2}=1.68, \psi_{1}=0.48, \psi_{2}=0.89, \zeta_{2}=2418$.

| $\beta_{2}$ | $\omega_{2}$ | $\eta_{2}$ |
| --- | --- | --- |
| 0 | 0 | 0.145 |
| 0.039 | 130617 | 343.706 |
| 0.380 | 3982266 | 687.910 |
| 3.705 | 121283345 | 1029.984 |
| 36.131 | 3692088103 | 1373.292 |
