# Supplementary material for: Host responses and viral traits interact to shape the impacts of climate warming on highly pathogenic avian influenza in migratory waterfowl
Source: PLoS Comput Biol. 2025 Oct 6;21(10):e1013451. doi: 10.1371/journal.pcbi.1013451 (PMC12513652; doi:10.1371/journal.pcbi.1013451)
Supplement: S4 Table — Binary outcomes variables (invasion probability and persistence probability) were evaluated with area under the receiver operating curve (AUC) and continuous variables were evaluated with relative root mean squared error (rRMSE). Metrics are provided for both the training and test data. (DOCX) [file pcbi.1013451.s023.docx]

**Host responses and viral traits interact to shape the impacts of climate warming on highly pathogenic avian influenza in migratory waterfowl**

Claire S. Teitelbaum, Michael L. Casazza, Cory T. Overton, Elliott L. Matchett, Diann J. Prosser

**S4 Table**: Model performance metrics for generalized additive models of HPAI outcomes. Binary outcomes variables (invasion probability and persistence probability) were evaluated with area under the receiver operating curve (AUC) and continuous variables were evaluated with relative root mean squared error (rRMSE). Metrics are provided for both the training and test data.

| **Outcome** | **Metric** | **Training** | **Testing** |
| --- | --- | --- | --- |
| Invasion | AUC | 0.956 | 0.961 |
| Persistence | AUC | 0.994 | 0.984 |
| Prevalence | rRMSE | 0.124 | 0.291 |
| Peak | rRMSE | 0.173 | 0.294 |
| Mortality | rRMSE | 0.042 | 0.085 |
