# Supplementary material for: Host responses and viral traits interact to shape the impacts of climate warming on highly pathogenic avian influenza in migratory waterfowl
Source: PLoS Comput Biol. 2025 Oct 6;21(10):e1013451. doi: 10.1371/journal.pcbi.1013451 (PMC12513652; doi:10.1371/journal.pcbi.1013451)
Supplement: Attachment — Submitted filename: PLoS_CompBiol_ReviewerResponse.pdf [file pcbi.1013451.s024.pdf]

May 16, 2025

Dear Dr. Scarpino,

We thank you and both reviewers for comments on the previous version of our manuscript and for the opportunity to submit a revised version. In response to reviewer and editor comments, we have made several major changes to model structure and have re-run all simulations. We have also revised large portions of the manuscript text to provide more justification and context for our approach. We explain these changes in detail below, but a summary of major changes includes:

1. ***Improved justification of homogeneous mixing***: The structure of our models is based on assumptions of homogeneous mixing among hosts at a single site. Because we did not have sufficient data to parameterize non-homogeneous mixing (e.g., contact networks), we instead have increased the realism of this assumption by:
    a. reducing the population size in the model from 10,000 to 5,000, which is a more realistic number of individuals to occur nearby year-round (i.e., a single sub-population).
    b. increasing the number of winter sites from one to three, with movements among sites throughout the winter. This change relaxes the homogeneous mixing assumption by allowing contact only among individuals at the same sub-site.
    c. reducing the contact/direct transmission rate at the breeding site to half of that at other sites. This change reflects that birds move less and interact with fewer other individuals while nesting.
2. ***Inclusion of cross-species transmission***: In the revised model, we incorporate elements of a multi-host system by modeling external input of virus into the environmental reservoir. This input occurs at a constant rate, so that it adds only one parameter to the model and does not require explicitly modeling a second species, but increases the realism of the model by allowing virus to be re-introduced to hosts from conspecifics or heterospecifics, even if it is temporarily eradicated from the focal host population.
3. ***Use of finer-scale climate data***: We updated our input climate data to a source with higher spatial resolution, which allows us to include local variation in climate conditions. In addition, instead of using smoothed temperatures across the year, we now use daily temperatures, which allow us to model viral decay in response to daily fluctuations in temperature. In addition, to streamline our analysis and results in an already-complex paper, we now use projections from a single climate change scenario.

With these changes, we believe we have addressed all reviewer comments, either through changes to the model or through additions to the introduction, methods, and discussion that provide more justification and context for our assumptions. Our primary results, which show multiple contrasting effects of climate change on avian influenza depending on climate impacts and host/viral traits, remain consistent despite these changes.

We look forward to hearing from you.

Sincerely,
Claire S. Teitelbaum (for all authors)

Editor Comments:

I agree with the reviewers that the authors provide an interesting modeling framework and application. I also agree that many of the modeling assumptions need strengthened justification and/or enhanced sensitivity analyses. In particular, I'd like to highlight the 1.) w/in site assumption of homogenous mixing

In the revised manuscript, we still assume homogeneous mixing within sites, but have made this assumption more realistic in three ways: by increasing the number of winter sites, by reducing the population size of modeled geese, and by decreasing contact transmission rates during the breeding season. We took this approach, rather than reformulating the models to include non-homogeneous mixing, because we did not have enough data to inform a realistic model of contact (e.g., contact network) or spatial proximity. Although our GPS telemetry sample size is significant, it still reflects a small proportion of the goose population. We now justify our choice of population size in the Methods (Line 187). We have also added a discussion of non-homogeneous mixing and its potential impacts on infection dynamics in the Discussion ("geese forage in groups of familiar individuals [85], which could result in slower or smaller outbreaks relative to equal contacts between all individuals at a site [86]", Line 523).

2.) fixed sites for migration (here I'm thinking of the potential importance of changing site availability under climate change)

We agree that changes in site use and spatial arrangement of sites is one potential response to climate change. Recent papers have explored this response in detail (e.g., Yin et al. 2022). In addition, we have little evidence for large-scale shifts in site use in this system in response to climate change, which would make it difficult to realistically model these changes (e.g., increases vs. decreases in number of sites used). In the revised manuscript, we include more detail on these prior studies and discuss possibilities for merging these approaches in future research ("the impacts of climate change on habitat availability for waterfowl can affect influenza dynamics [91], for example by concentrating animals at fewer sites [25], and future studies that incorporate both spatial and temporal changes in migration would be valuable", Line 532).

Yin, S., Xu, Y., Xu, M., de Jong, M. C., Huisman, M. R., Contina, A., ... & de Boer, W. F. (2022). Habitat loss exacerbates pathogen spread: An agent-based model of avian influenza infection in migratory waterfowl. PLoS Computational Biology, 18(8), e1009577.

3.) the gradual nature of temperature change and using temperature as the key driver of climate change

We have modified the input temperature variables in our models so that temperature fluctuates daily, which makes temperature change less gradual than in the prior version of the manuscript, which used a smoothing function to estimate daily temperatures (see revised Figure 1). Our use of temperature as a key variable is based on our knowledge of

its impacts on both goose migration behavior and viral kinetics. Although both bird migration and viral dynamics can and do respond to other variables (e.g., precipitation), we have much less information about the consistency of these responses, and much less quantitative data for parameterizing these responses in our models. For example, prior empirical work in this system shows a relationship between goose migration phenology and temperature, but we have no such documented relationship for other climate variables. Temperature is also the climate variable for which we have the most accurate and reliable projections. We therefore continue to focus on temperature as the main variable underlying climate change.

4.) the evolutionary dynamics of host/virus

Evolution, and viral evolution in particular, is a very important aspect in the waterfowl-influenza system. As this paper is already so complex, modeling evolution explicitly is beyond the scope of this analysis. However, by performing extensive sensitivity analysis over a range of viral traits for both low pathogenic and highly pathogenic influenza viruses, we address potential consequences of novel viral traits (e.g., increases or decreases in virulence). In the revised manuscript, we have added more discussion of the importance of viral evolution. We have also edited the manuscript to more clearly link our exploration of viral traits to viral evolution (Introduction: "understanding the roles of HPAI viral traits and host responses for HPAI dynamics in wild bird populations can help us forecast how these dynamics might continue to change in the future, as these viruses continue to evolve and adapt to new hosts, new environments, and new transmission modes", Line 56) (Discussion: "HPAI viruses are continually evolving in natural systems [96]. Although our models considered a range of viral traits that represented potential outcomes of this evolution, future models that explicitly incorporate the processes of mutation and selection could provide more insight into the likely trajectory of these viruses, in particular how they might adapt to continuing shifts in host ecology and environmental conditions," Line 556)

5.) the identifiability of the environmental transmission component.

We have updated our simulations to include HPAI strains (i.e., parameter sets) where transmission is entirely environmental (β=0) and those where it is entirely direct (ω=0). Results from modeling these strains supports our conclusions about the relative importance of each of these transmission modes for HPAI invasion, prevalence, and persistence (Line 315, Line 373). These results are consistent with our general interpretation of the role of environmental transmission for HPAI dynamics.

From my perspective, addressing these concerns will require additional simulations and likely cannot be fully addressed solely by adding caveats to the discussion. Each of the reviewers also provided a number of additional comments/suggestions, which I believe will strengthen the manuscript. A few I'd like to highlight are 1.) moving some of the figures from the supplement to the main text, 2.) streamlining and adding clarifications to the introduction, and 3.) ensuring that terms are clearly defined and reflect standard usage in the field

We have run all simulations again to address reviewer comments. Please see responses below to individual reviewer comments regarding organization and terminology.

Reviewer #1:

Dear Authors,
Thank you for submitting your manuscript on the impacts of climate change on HPAI dynamics in migratory waterfowl. This is a critical area of research, and your study offers valuable insights into how viral transmission traits and environmental changes might interact to influence disease dynamics. However, after reviewing the study, I believe there are several areas where revisions and additional clarifications would strengthen the manuscript.

Abstract:
• The abstract starts with a broad statement about emerging infectious diseases, which is somewhat generic. Although it sets the stage, it would be stronger if it immediately focused on the topic at hand (avian influenza and climate change).

While the reviewer's point is well taken, we prefer to maintain broad statements at the beginning and end of the abstract because this modeling approach is applicable to other diseases as well, and we hope the manuscript is of interest to readers who do not study avian influenza.

• The mechanistic model is briefly mentioned without enough context. It's unclear what specific features of the model were novel or important to the study.

This model itself was not particularly novel; it was modified from published literature. It is the application of the model that is novel, to apply it to HPAI (instead of LPAI) and incorporate changes in migration phenology. We have edited the abstract to state that we "modify an existing mechanistic model."

• While the focus is on transmission traits and climate change, the direct impact on migratory waterfowl populations is glossed over. The abstract should state more explicitly how these changes affect the overall ecology or population dynamics of waterfowl.

We agree that mortality is probably the most important impact we measure in this model. We have added a sentence to the abstract to describe the range of impacts on the host population (ranging from no mortality to 100% mortality, depending on the parameterization).

• The abstract ends on a general note about interactions between host and viral ecology. This could be more impactful by stating concrete findings or practical implications for wildlife management or conservation.

We have replaced this final sentence with a statement about future directions and added a new sentence that summarizes our findings ("Together, these results suggest that climate

change is likely to increase the impacts of HPAI on waterfowl populations if HPAI relies strongly on direct transmission and birds advance their spring migration.")

• Terms like "most important factors" and "slightly increases" are vague. The magnitude of the effects should be stated, even if approximate, to provide a clearer idea of the findings.

We no longer use these phrases in the abstract and have focused the abstract more clearly on our results.

• The description of climate change impacts (e.g., warmer environments reducing viral survival) is not sufficiently detailed. The mechanisms by which climate change affects viral transmission should be clearer, especially considering the direct and indirect pathways mentioned.

We have edited this section of the abstract to include more detail on the phenomena and mechanisms ("we found that a 9-day advancement in spring migration timing increased the duration and decreased the peak size of HPAI outbreaks by increasing time birds spend at their breeding grounds, leading to higher mortality").

• While the focus on transmission traits (e.g., contact and shedding rates) is important, the abstract does not address whether other potential factors (e.g., genetic adaptations or immunological responses) were considered.

We have added a sentence in the abstract to highlight key questions for future work that are not addressed in this model ("Future work could further integrate host-viral co-evolution and other climatic changes (e.g., salinity, humidity) to provide a precise predictions of how HPAI dynamics are likely to change in the future"). We have also added reference to other parameters that are included in the model (Line 11-12).

Introduction:

• The introduction starts by discussing "emerging infectious diseases" in general, which dilutes the focus. While it's good to mention global relevance, the introduction could benefit from narrowing down to avian influenza and climate change impacts earlier to engage the reader with the study's specific objectives.

We have shortened this introductory material and have brought information about HPAI into the first paragraph.

• The introduction briefly mentions that climate change affects disease transmission via species distributions, but it lacks detailed exploration of the mechanisms by which climate change interacts with avian influenza transmission, persistence, or virulence.

The second paragraph of the introduction (Line 60-71) discusses the role of temperature for avian influenza transmission and persistence via both environmental decay and species distributions.

• The literature cited (e.g., on climate change impacts or avian influenza dynamics) is presented without much critical analysis. For instance, how the cited models or studies shaped the current study or how gaps in those studies are being addressed is not discussed.

> We have added a sentence to the introduction that clarifies that this paper adds to this literature by producing quantitative predictions for the impacts of climate change on avian influenza in wild birds ("despite these many hypothesized impacts of global change on wild bird populations, we have little empirical evidence and few quantitative predictions for how climate change will impact avian influenza dynamics in wild birds (but see [25,31])," Line 80).

• The introduction cites general reviews (e.g., [4–6] on climate change effects) without discussing specific examples or how the present work builds upon or diverges from these previous studies.

> These cited reviews provide extensive evidence that climate change can impact infectious diseases. This study focuses on a particular study system and question, and the rest of the introduction more clearly describes how this study builds on prior work on avian influenza (e.g., inclusion of climate change).

• The decision to use a mechanistic model and the reasons for choosing specific parameters (e.g., migration phenology) are not well explained. There is no discussion of why greater white-fronted geese were chosen as the focal species and how representative they are of waterfowl or other species affected by HPAI.

> We have added a statement in the Introduction to justify the use of migration phenology as the host response to climate change ("there is ample evidence of temperature-related changes to migration phenology over the last few decades [36,37]," Line 93). The introduction also discusses the choice of greater white-fronted geese as a model system (Line 87-95) and migration responses to climate change more generally (Line 67-69).

• The introduction does not clearly state the hypotheses being tested or the expected outcomes. This leaves the reader guessing as to the study's intended contributions.

> We have added hypotheses about the interactions between climate change impacts and HPAI traits as the last sentence of the introduction ("We expected that climate change would reduce HPAI impacts for environmentally transmitted strains when climate change impacts viral decay, and would increase HPAI impacts for directly transmitted strains when climate change impacts migration phenology," Line 99).

Methods:

• The SIR model assumes homogeneous mixing within sites, which oversimplifies real-world conditions where migration and contact rates are likely heterogeneous. The validity of this assumption should be discussed, especially in relation to waterfowl's complex social and migratory behaviors.

In the revised manuscript, we still assume homogeneous mixing within sites, but have made this assumption more realistic in three ways: by increasing the number of winter sites, by reducing the population size of modeled geese, and by decreasing contact transmission rates during the breeding season. We took this approach, rather than reformulating the models to include non-homogeneous mixing, because we did not have enough data to inform a realistic model of contact (e.g., contact network) or spatial proximity. Although our GPS telemetry sample size is significant, it still reflects a very small proportion of the goose population. We now justify our choice of population size in the Methods (Line 187). We have also added a discussion of non-homogeneous mixing and its potential impacts on infection dynamics in the Discussion ("For example, geese forage in groups of familiar individuals [85], which could result in slower or smaller outbreaks relative to equal contacts between all individuals at a site [86]", Line 524).

• The model distinguishes between direct and environmental transmission but doesn't explain the criteria for classifying transmission modes in specific environmental contexts. This weakens the understanding of how robust the model is to various ecological settings.

We do not fully understand what is meant by "classifying transmission modes in specific environmental contexts." However, in the revised manuscript, we have added parameter sets with only environmental transmission and only direct transmission, which allows us to more clearly separate the roles of these two processes. These results are described at Line 315 and Line 373. We have also modified our method for selecting HPAI strains in our climate-change analysis, such that we now include strains with only environmental transmission and only direct transmission. Once again, this method allows us to explore the sensitivity of each transmission mode to temperature changes. These results are shown in Figure 6.

• The model primarily focuses on traits like shedding and contact rates, which oversimplifies the full spectrum of viral dynamics. Other factors, such as viral mutation rates, antigenic drift, or immune system responses, are not considered, limiting the model's ecological validity.

As discussed above, although many other traits are important for HPAI dynamics, explicitly modeling viral evolution is beyond the scope of this paper. To address this comment, we have made several changes to the manuscript:

- We address in the introduction that "HPAI viral traits *and host responses*" together determine HPAI dynamics (Line 56).
- We explicitly state that no evolution is included in the model ("We assume that viral and host traits are fixed over the course of a single parameterization of the model (i.e., no evolution occurs)," Line 127)
- We note that the traits we model can represent host as well as viral traits ("Although we refer to these parameter sets as "strains," they could represent host as well as viral traits (e.g., host immune function impacts infection duration)," Line 213)
- We discuss the importance of viral evolution in the Discussion ("Further, HPAI viruses are continually evolving in natural systems [96]. Although our models considered a range of viral traits that represented potential outcomes of this evolution,

future models that explicitly incorporate the processes of mutation and selection could provide more insight into the likely trajectory of these viruses, in particular how they might adapt to continuing shifts in host ecology and environmental conditions," Line 556).

• The method relies heavily on viral decay rates being parameterized based on temperature, but this ignores the influence of other environmental factors such as humidity, UV radiation, or host population density, which can also significantly affect viral persistence.

As described above, our use of temperature as a key variable is based on our knowledge of its impacts on both goose migration behavior and viral kinetics. Although both bird migration and viral dynamics can and do respond to other variables (e.g., precipitation), we have much less information about the consistency of these responses, and much less quantitative data for parameterizing these responses in our models. Experiments focusing on humidity and UV radiation have, to our knowledge, all focused on HPAI decay in chicken litter, and it is unclear how these experiments would translate to "real-world" conditions. We continue to focus on temperature but address these other environmental conditions as opportunities for future research (Line 502-506; Line 516-518).

• The choice of viral decay rate parameters from reanalysis products (MERRA-2) is presented without sufficient validation or comparison to empirical field data. The reliance on reanalysis data is a potential limitation, as such data may not accurately capture micro-environmental conditions where viral transmission occurs.

We agree that these products do not – and cannot – capture microenvironments, and that substantial variation exists within a site. We have made several changes to the manuscript to address this concern:

- We now use the ERA5-Land reanalysis product, with a spatial resolution of 11 km, in place of the MERRA-2 reanalysis product (spatial resolution 50 km). This change increases our ability to capture within-site variation in climate conditions.
- We use daily temperature estimates from the reanalysis, rather than smoothed functions of temperature, in the models. This change reflects finer-scale temporal variation in temperature, increasing the realism of the model.
- We discuss the importance of micro-habitat variation in the Discussion ("It is possible that these results would differ under different climate scenarios or with the inclusion of fine-scale data on microhabitats (e.g., temperature stratification in water, fine-scale variation in temperature between sun and shade)," Line 543).

• The model's assumption of unidirectional migration and fixed seasonal sites may not reflect the dynamic and variable migration behavior of geese in response to changing environmental conditions. This could result in inaccurate predictions of virus spread and persistence.

We have added additional text in the Introduction and Discussion to acknowledge that migration phenology is not the only mechanism by which climate change could impact avian influenza dynamics:

- Introduction: "Models show that habitat loss can promote influenza transmission in migratory hosts by increasing group sizes at remnant sites [25,26]," Line 73
- Discussion: "migration could adapt differently than observed, for example by advancing more than expected or by altering the number or spatial configuration of sites," Line 545.

We continue to focus on migration phenology because it is the most consistent, well documented change in goose migration behavior in response to climate change. Additionally, other studies (e.g., Yin et al. 2022) have explored questions related to habitat loss in detail.

Yin S, et al. Habitat loss exacerbates pathogen spread: An Agent-based model of avian influenza infection in migratory waterfowl. PLoS Computational Biology. 2022;18: e1009577. doi:10.1371/journal.pcbi.1009577

• Although the paper mentions changes in migration timing due to climate change, there is no consideration of potential mismatches between migration and food availability, which could drastically alter infection dynamics.

This is an excellent point, especially if hosts fail to sufficiently adapt their migration phenology to climate change. We have added this point to the Discussion: "impacts might also differ… if phenological mismatch due to climate change limits hosts' abilities to respond to infection [95]" (Line 548).

• While the methods mention stochastic processes, it is unclear how much variability was introduced into the model and whether sensitivity analyses were performed to assess how robust the results are to parameter uncertainty.

Models were simulated stochastically such that the probability of any event (e.g., infection, recovery, movement) occurring in each timestep was probabilistic. These probabilities were based on fixed parameters; the parameters themselves were not modeled stochastically, so parameter uncertainty was not incorporated into the stochastic nature of the simulations. We have added text in the methods to clarify: "In this stochastic framework, rate parameters (e.g., mortality rate) are used to determine the probability of an event occurring in each time step; rate parameters themselves remain fixed (or vary deterministically) over the course of a simulation," (Line 175-178).

In addition, please note that the first two sets of simulations (LPAI-only and LPAI-HPAI) were essentially large sensitivity analyses, the results of which analyze how results change as a result of parameter uncertainty.

• The parameters used for migration and infection dynamics (e.g., decay rates, contact rates) are introduced with minimal justification or reference to empirical studies, making it difficult to assess whether the model is biologically realistic.

References to empirical and modeling studies are included in Table S1. We have added additional references to this table. In the prior version, we had included only references that represented the minimum or maximum value of the range. We have also added a sentence in the methods to clarify that some parameters have well defined ranges from empirical studies, while others do not (Line 189-192). In addition, we have now include reference to a recent review of challenge studies that estimate many of these parameters (Gonnerman et al. 2024). Finally, following the suggestion of Reviewer 3, we have expanded on parameter uncertainty with a full paragraph in the discussion (Line 485-505).

Results

• The study modeled 10,000 combinations of viral traits but without clear justification of how these traits realistically reflect natural viral diversity or evolutionary dynamics. For example, the models treat viral shedding rate (ω2) and transmission rate (β2) as independent, yet in reality, viral strains with higher shedding may also exhibit lower transmission due to immune response or other compensatory factors. This oversimplification may lead to unrealistic invasion outcomes.

This is true; some outcomes of the model are unrealistic, which is why we performed sensitivity analyses for LPAI and HPAI before selecting parameter values for the climate change analysis. We have added text to the methods to acknowledge that parameters could be correlated in reality: "This method assumes that parameters are independent; although some parameters might be related in reality (e.g., shedding rate and contact transmission rate [59]), this approach allowed us to explore the effects of each HPAI trait individually," Line 219).

• There is no discussion on the empirical basis for the range of viral traits (e.g., direct transmission rate or mortality rates). Without proper calibration against real-world data, the modeled viral strains may not represent the actual behavior of HPAI, making predictions less reliable.

Please see response above about parameter ranges, which are justified in Table S1.

• The results indicate that direct transmission rate (β2) was the most influential factor for invasion success. However, the model does not explore how environmental factors or host behavior might impact transmission in more complex scenarios (e.g., within-season migration patterns, mixed populations). This reductionist approach undermines the ecological complexities of migratory populations, where contact rates and social structure could play a significant role in disease spread.

We agree that this model represents as simplified version of this natural system. In the revised version of the manuscript, we have added two additional parameters to the model, which relax these simplifications. First, we include within-season movement during winter, so that geese move continuously among three winter sites. Second, we add an external input of virus into the environment, which represents infection of other species or populations. In addition, in the revised manuscript, we now quantify variable

importance, which allows the reader to compare the relative influence of each parameter on invasion success and subsequent outcomes (Figure 2). This addition clarifies that, although direct transmission was the most important parameter for invasion success, other parameters were also important.

• Cross-immunity ($\psi 1$) is mentioned as having limited influence on invasion probability, but this contradicts other studies that suggest prior immunity can strongly influence viral transmission dynamics. The limited exploration of cross-immunity seems like a missed opportunity, especially since cross-immunity may be a key factor in HPAI evolution. Additionally, the study focuses solely on the migratory goose population, yet HPAI is known to infect multiple hosts, including non-migratory species. The absence of a multi-host model could result in underestimating the risk of persistent HPAI transmission and endemicity, particularly in mixed species environments like wetlands.

The reviewer is correct that this was a surprising finding! A prior study that used and developed this model explored the role of cross-immunity for HPAI dynamics (Brown et al. 2014). Surprisingly, they also found that cross-immunity was not necessary to generate the HPAI dynamics observed in empirical data. These same authors also explored a multispecies system in detail (Brown et al. 2013). To address these concerns we have:

- added more detail about prior uses of this model in the Methods (Line 107).
- added an external environmental input of virus into the model for both LPAI and HPAI, to include the potential influence of heterospecifics on viral persistence.

Brown, V. L., Drake, J. M., Stallknecht, D. E., Brown, J. D., Pedersen, K., & Rohani, P. (2013). Dissecting a wildlife disease hotspot: the impact of multiple host species, environmental transmission and seasonality in migration, breeding and mortality. Journal of the Royal Society Interface, 10(79), 20120804.

Brown, V. L., Drake, J. M., Barton, H. D., Stallknecht, D. E., Brown, J. D., & Rohani, P. (2014). Neutrality, cross-immunity and subtype dominance in avian influenza viruses. PloS one, 9(2), e88817.

• The study defines an invasion as ≥100 infections, but this threshold is arbitrary and may not correspond to a meaningful epidemic. In smaller populations, 100 infections could represent a significant fraction, while in larger populations, it may be negligible. This rigid threshold could skew the interpretation of results, especially when considering migratory populations where flock sizes fluctuate seasonally.

It is true that the threshold is arbitrary. In this model, 100 infections was a breakpoint in the data that corresponded to true invasion and persistence. In other words, if a strain caused at least 100 infections, it was likely to cause many more. We have clarified this in the text: "defined as causing >100 infections, a breakpoint in the data above which strains were likely to cause more infections and persist for longer" (Line 251). We have also

updated Figure S4 to show the relationship between peak infections and mean infection prevalence. We are do not advocate that this threshold be used in real-world systems.

• While the model considers environmental persistence ($\eta 2$), the results show weak effects of environmental persistence on viral outcomes. This could be due to oversimplified assumptions regarding how temperature influences viral viability in the environment. The model does not account for factors like water salinity or UV exposure, which are known to affect virus survival, especially in aquatic habitats. This could lead to an underestimation of the role of environmental reservoirs in HPAI dynamics.

In the revised version of the manuscript, we have broadened the range of the environmental persistence parameter. Previously, we based our parameter range on prior studies of LPAI viruses; however, because our model seeks to explore a broad range of potential outcomes, this comment highlighted that it is possible that novel strains or other characteristics could push these parameters beyond results from laboratory experiments. This broader range increased the model's sensitivity to environmental persistence of the virus, which we discuss in more detail in the revised manuscript (Line 366-375, Figure 5).

As we detailed in our comments to the editor above, while we agree that other factors including humidity, UV radiation, and salinity can affect viral persistence, we continue to focus on temperature because of its clear connection to climate change and the paucity of literature on host and viral responses to these other environmental parameters in natural systems.

• The study's approach to temperature sensitivity lacks granularity; it uses broad categories (e.g., temperature-sensitive strains) without detailing how small temperature changes influence viral persistence. The nonlinear effects reported should be more rigorously explained.

In the revised manuscript, we include a more detailed study of temperature sensitivity. In addition to the broader parameter range in our HPAI sensitivity analysis (see previous comment/response), we now consider a range of temperature sensitivities in our climate change analysis (i.e., five values of η_2 in place of a classification). We also now describe the interactions of η_2 with other model variables (notably shedding rate) in the results (Line 366-375, Figure 5).

• The results mention that persistence duration was insensitive to direct transmission rates but was influenced by mortality rates ($\nu 2$). This finding contradicts well-established principles in disease ecology, where higher transmission often leads to longer persistence due to sustained chains of infection. The lack of discussion on why direct transmission did not significantly impact persistence is a major weakness, as it leaves a key question about disease longevity unresolved.

In the revised manuscript, our quantification of variable importance shows that direct transmission rates were important for persistence, as were mortality rates or infection

duration. We have reworded the results to clarify that direct transmission rates were still important.

• The study models its outcomes using generalized additive models (GAMs), but it does not provide enough detail on model diagnostics, such as goodness of fit or validation against independent data sets. Without knowing how well the model predicts observed outcomes, it is difficult to assess the accuracy of the predictions. Additionally, GAMs can obscure underlying causal relationships by overfitting the data, especially when nonlinear effects are involved.

Thank you for this comment. In the revised manuscript, we provide more detail in the methods about the diagnostics used to evaluate model fit (Line 272-277) and report these diagnostics in the results (Line 306-308, Table S4). Briefly, we used model evaluation metrics (AUC and rRMSE) to evaluate model fit on the training data, as well as on independent validation data. In addition, we used multiple methods to ensure that our GAMs were not overfit, including limiting the number of knots for each variable and using integrated model selection (Line 269-271).

Discussion

• The discussion suggests that climate change will have opposing effects on HPAI dynamics depending on viral persistence or migration timing. This binary framing oversimplifies the complex interaction between climate, bird behavior, and virus ecology. For instance, it fails to account for how climate change might impact not just the timing of migration but also the geographic range of migratory species, potentially introducing HPAI to new hosts or ecosystems.

It is true that there are myriad ways that climate change could affect HPAI dynamics. In this study, we focus on two (migration phenology and viral persistence), which is why the discussion is focused on these two responses. In the introduction, we describe more ways in which climate change can affect host and viral ecology, and in this first paragraph of the introduction, we acknowledge that this study represents a "simplified version" of a natural system (Line 454, Line 518).

• Additionally, the discussion assumes that warmer climates will uniformly reduce viral persistence, but this may not hold in regions where other factors, like humidity, rainfall, or habitat degradation, might maintain environmental viral reservoirs despite increased temperatures.

In the discussion, we acknowledge that other environmental conditions impact viral persistence, and that these conditions are likely to be impacted by climate change. We have added more text to explicitly state that "In addition to rising global temperatures, climate change is also impacting precipitation regimes, habitat quality, and other environmental parameters, whose effects on viral decay rates could differ from those of temperature" (Line 515), in addition to the existing text calling for more experimental work quantifying responses of AIVs to pH and salinity (Line 517).

• The discussion briefly acknowledges that multi-host dynamics play a role in viral persistence (lines 348–350), but this point is underdeveloped. Given that influenza viruses often spill over

between species, the model's failure to incorporate multi-host interactions limits its applicability. This omission is particularly relevant for understanding how species with different migration patterns or immune responses (e.g., ducks, shorebirds) could influence the overall persistence and spread of HPAI.

> In the revised model, we have added an external input of virus into the environment to model the contribution of heterospecifics to HPAI dynamics. This parameter was important for determining HPAI infection prevalence and mortality, especially when HPAI persisted for long periods in the environment. We now describe this result in the text (Line 371). In addition, we provide more detail on why multi-species modeling is important in the discussion (Line 478-484).

• The authors discuss how shifts from direct to environmental transmission could promote longer outbreaks or HPAI endemicity, but they do not explore the evolutionary implications of this shift. For example, will future strains become more temperature-resistant, or could selection pressure favor strains with higher transmission rates? A more detailed evolutionary perspective would strengthen the argument about how HPAI dynamics might change in response to environmental or host changes.

> This is true; although our model suggests that these traits would be favorable, we have no evidence for the ability of the virus to actually achieve any combination of traits. Because modeling this evolution (and constraints) is beyond the scope of the current study, we propose it as an avenue for future research: "Future studies that consider constraints on viral evolution (e.g., tradeoffs among traits) could further clarify how selection pressures, viral physiology, and environmental conditions could interact to promote or constrain environmental transmission rates of HPAI viruses" (Line 469-472).

• The discussion does not adequately address the study's limitations, such as the use of simplified host demographics (ignoring age structure and interspecies interactions), the arbitrary invasion threshold, or the potential bias in model parameter ranges. Without explicitly acknowledging these weaknesses, the predictive power of the model is overstated, reducing confidence in its utility for forecasting real-world HPAI dynamics.

> We list many of these limitations in the discussion (some in the original version of the manuscript; some added in revision). We respectfully disagree that the predictive power of the model is overstated; we do not intend for this model to provide quantitative predictions, nor does the paper imply that our results should be used as predictions. Instead, we conclude by stating that our modeling efforts advance our understanding of how multiple ecological processes fit together (i.e., advancement of theory). While these findings have implications for forecasting down the road (e.g., by identifying parameters and processes that should be included in forecasts), this study does no forecasting itself.

• While the authors call for more experimental studies on AIVs (lines 367-372), they do not sufficiently integrate existing empirical data into their model. For example, temperature sensitivity and shedding rates are mentioned but not well-supported by field or lab-based studies.

The model's predictions would be more credible if it were more closely tied to real-world observations of HPAI outbreaks.

> Our study incorporates empirical data in three ways: defining parameter ranges (see Table S1), validation of LPAI dynamics, and climate data. Although we agree with the reviewer that using observations of HPAI outbreaks would be ideal, the existing empirical data are unfortunately insufficient for model validation. For example, we have very limited information about mean infection prevalence, mortality impacts, or peak (initial) infection prevalence in wild goose populations. We identify this validation as a need for future models in the Discussion: "These models would be strengthened by validation with empirical data on HPAI infection prevalence and its demographic impacts in goose populations, especially as surveillance efforts continue" (Line 529).

Reviewer #2

The study by Teitelbaum and co-authors presents a timely and well-executed study that advances understanding of avian influenza transmission under climate change scenarios using Greater White-fronted Geese as a model organism. Using a mechanistic model that integrates migration phenology and HPAI infection dynamics, the authors explore critical aspects of transmission that represent large knowledge gaps for the influenza field. The integration of direct and indirect effects of climate change into the compartmental model is particularly novel and insightful. The study lends support to the hypothesis that climate change will have opposing effects on HPAI transmission in migratory waterfowl due to reduced viral persistence in a warming climate, but also increased mean prevalence and mortality related to advancing spring migration. This Overall, I thought the study was thoughtful in paying attention to biologically-relevant variables and parameters that drive influenza infection at ecological scales, and am excited to see the impact that this work will have on the influenza and disease ecology field at-large.

I have a few recommendations to help strengthen the study, mostly with the goal of embedding the viral kinetics in empirical data derived from field or experimental studies:

> Thank you for this summary and positive assessment.

- The consideration of two influenza phenotypes: LPAI and HPAI, and their cross-reactivity is insightful and demonstrates the authors have current working knowledge of influenza biology and the relevance of antigenically different strains. The endemic circulation of LPAI has been postulated as a major factor explaining the heterogeneity in disease outcome within and between avian hosts during the current HPAI outbreak (although never explicitly tested). However, cross-reactivity is treated as a binary variable: low and high, which is an over-simplification that may undermine the relevance of the model. For example, gulls in which H13 and H16 circulates endemically would offer little or no immunological protection against HPAI H5. I wondered if this could be characterized as a continuous trait measured by antigenic distance of the HA gene segment. Pairwise interactions between the 16 LPAI subtypes vs 1 HPAI subtype could be quantified and used to derive an underlying distribution of cross-protective immunity that would add resolution to the model, and make the results more biologically meaningful.

We agree that cross-immunity is a very important component of the AIV system. First, we should clarify that cross-immunity is not treated as binary, but instead takes a range of values between 0 and 1. We have re-read the manuscript and unfortunately were unable to locate the section of text that might have caused this misunderstanding. We do consider two components of immunity (protection from infection vs. protection from mortality), based on experimental studies that show that these two types of protection vary, sometimes independently. In addition, we agree that the reviewer's idea about antigenic distance is an interesting one; however, we are unaware of systemic information about the cross-reactivity between each HA type and H5N1 that could be used to parameterize this mechanistic interaction. Instead, we believe that using the full range of cross-immunity covers all possible combinations.

- Climate change is modeled as the mean annual temperature across each of the 4 sites of the annual cycle. This approach treats climate change as a gradual temperature change, without considering the increased variability in temperature at each of the 4 sites. Could the authors comment on how accounting for the two temporal scales of climate change - rapid (extreme temperature fluctuations) and gradual (mean temperature increases) might change the outcome of the model. For instance, the process of freeze-thawing can lead to degradation of the viral particle, which may further reduce viral persistence especially at habitats that exist on or near the 0 degree threshold.

In the revised manuscript, we now include day-to-day fluctuations in temperature to account for increased variability in temperature with climate change. As the reviewer notes, climate change is associated with a change in variance as well as in mean temperature. We have made several changes in response to this comment:

- In the previous version of the manuscript, we had used a smoothed function of temperature to estimate daily temperature; in the revision, we include observed (historical) or modeled (future) daily temperatures (see Figure 1).
- In the previous version of the manuscript, we used mean temperature as a proxy for climate change in our data analysis. In the revised manuscript, we use instead use calendar year (i.e., 2020 vs. 2095), so there is no need to calculate a mean.
- We have added freeze-thaw cycles as another climate-related change that could impact viral survival in the Discussion (Line 542).

Minor comments:

- The use of the term 'pathogen invasion' isn't commonly used in influenza literature, or at least not to my knowledge. I can see it relates to the Rohani paper from 2006. I'm guessing this is functionally the same as introduction of the virus into a new population. Might be worth clarifying that in the introduction.

Thank you for this comment; although this term is relatively common in ecological epidemiology but may not be familiar for our entire audience. "Invasion" refers not just to introduction but also to establishment in a population. We have replaced the word

"invasion" with "establishment" in the abstract and defined it as first use in the introduction (Line 82).

Reviewer #3

This paper provides a comprehensive assessment of the role that high pathology avian influenza (HPAI) could play in the population dynamics of a migratory bird. Using a simulation approach, the different intrinsic traits of the HPAI strain, changes in climate, and migratory behaviors of the species can be assessed in concert to assess these factors for overall importance and plan for likely future scenarios. Overall, the modeling approach is robust, well-documented, and well-executed. This was a significant effort that was undertaken with a consistent logic and purpose.

Thank you for this positive assessment.

There are some areas this paper can improve upon. This paper has a fair bit going on in it with all the different scenarios tested and communicating the results of this paper is challenging. With so many scenarios tested and simulations run, there are some difficulties in communicating complex and interacting results. I would prefer to see more figures in the main paper to help the reader understand the patterns. I spent a fair bit of time in the SI when reviewing the paper and I found the figures there were very helpful to understanding the results. I would bring in a few of the SI figures into the main paper. S7 provides overall summary statistics for the effort that would be helpful for the readers to see, and S9 provides support for a key component of the paper. Maybe even Figure 4 is useful for communicating the overall rates of strain invasions. But more information and data visualization are needed in the main paper. Further, the main paper figures also needed some updates. I provide more details in the line-by-line comments there.

We agree that the study is very complex; indeed, we found it difficult to figure out which results to include in the main text. In the revised version, we have reworked most figures and have included more results in the main text (as well as in the SI). In addition to the updated figures that replaced already-existing figures, we now include:

- A visualization of variable importance (see next comment and response; Figure 2)
- Histograms of HPAI outcomes (previously Figure S7, now Figure 3)
- Plots of the impacts of temperature sensitivity on HPAI outcomes (previously Figure S9; reformatted and now Figure 5)
- Time-series of climate change impacts (Figure S3)

The second larger point is what you should do for all parameters and scenarios that were tested. This paper has some issues with information overload, and a few changes could help the reader out. First, I think a summary table or figure describing the overall importance of each parameter would be helpful. I realize this would be challenging given all the complexities here, but something to provide an overall assessment of significant relationships or interactions across the parameters would go a long way.

We have created a metric of variable importance that is now included in the manuscript (see new Figure 2). This metric quantifies importance for each parameter for each

outcomes, and we include it for each parameter and pairwise interaction. We think this addition has helped streamline the results so that readers can clearly see a summary of results in one place; thank you for the suggestion. We also note in the figure legend that variable importance is related to parameter uncertainty (see next comment/response).

Furthermore, there are knowledge gaps in the parameters chosen for this paper. Some parameters are well-known, and others are not. More detail on when a parameter value incorporated into the simulation study is precautionary or well-supported by the literature would help direct the readers' attention. Further, you could combine your assessments of current knowledge with model sensitivity across all the parameters to determine which values we need more information on. These types of studies are only as good as the data that go into them, and directing future research to areas where more accuracy is required would be a valuable contribution.

Even with a few issues, I found this paper very well done and interesting to read. Thanks for the contribution.

We have added a paragraph to the discussion that expands on questions of parameter uncertainty (as suggested below in line-by-line comments). This paragraph highlights that some parameters are difficult to define by nature (β), but that our results highlight others as potential targets for future empirical quantification (Line 485-505).

Line-by-line comments:

26-27. This sentence is a bit awkward. What is ‘those’ specifically? Also, pointing out that more deadly outbreaks can cause significant mortality doesn’t seem helpful or specific.

We have replaced this sentence with: “these viruses have more severe impacts on wild bird populations when they are more easily transmitted, infection lasts longer, and prior infection provides little immunity.”

197. SSP 3-7.0 is on the higher end of climate predictions, I don’t think it can be solely described as moderate.

In the revised manuscript, we now use only SSP 5-8.5 (an “extreme” scenario), because it provides the clearest exploration of a “what-if” scenario for future climate change. This scenario is also one with the clearest increases in temperature variance in addition to changes in the mean. We define the scenario as “severe” in the text (Line 94, 242).

217. I would change this to ‘...all could be defined on the interval of [0, 1];...’

We have edited the text as suggested.

247. Are transmission rates from 0.1 to 1 considered low to intermediate? It should be something like ‘low to maximum’ at least. This doesn’t seem like a useful category of values to me.

We agree that this classification was not particularly meaningful, especially since β itself does not have an intuitive interpretation. The point of this statement was to identify a range of values at which β was low enough that other parameters still had some influence. Instead of "low to intermediate," the revised manuscript describes this range as "When direct transmission rates were low enough that invasion probabilities were <1 ($10^{-1.5} < \beta_2 < 10^{-0.5}$)" (Line 317).

309. I think the different symbols and transparencies are difficult to read in Figure 3. The y-axis labeling is confusing, and it's difficult to parse what matters. I would try changing the symbology or a different type of figure altogether.

We have fully revised this figure in the updated manuscript (see new Figure 6). The y-axis now measures expected change over 75 years rather than change per degree Celsius, which we find to be a more intuitive metric. We also include fewer HPAI strains in this figure (with full results in SI) to avoid needing to use different symbols and transparencies and accordingly now use a bar plot instead of points.

354. This finding should be more clearly shown in the main paper.

This result was weaker following revisions to the methods (in particular, because we now use daily instead of smoothed temperature data), so this paragraph has been removed.

369. This sentence seems like it should be an entire paragraph in the discussion. Given that you are using fairly wide ranges for many of the variables because of uncertainty in model parameters, can the parameter inputs be evaluated in terms of prior knowledge? Table S1 covers how each of the inputs was derived, but it would be useful to know which parameters had limited knowledge and which were more strongly informed. Further, this could be carried over to something like Figure 2, where the more likely parameter values could be identified to direct our attention.

We now include a paragraph in the discussion that provides more detail on parameter ranges, the limitations in defining them, and suggested priorities for future work in this area (Line 485-505).

389. You don't need 'historically' in this sentence.

Removed.

Journal Requirements:

1) We have noticed that you have uploaded Supporting Information files, but you have not included a list of legends. Please add a full list of legends for your Supporting Information files after the references list.

The list of supplementary legends is now included in the main text.

2) Some material included in your submission may be copyrighted. According to PLOSu2019s copyright policy, authors who use figures or other material (e.g., graphics, clipart, maps) from another author or copyright holder must demonstrate or obtain permission to publish this material under the Creative Commons Attribution 4.0 International (CC BY 4.0) License used by PLOS journals. Please closely review the details of PLOSu2019s copyright requirements here: PLOS Licenses and Copyright. If you need to request permissions from a copyright holder, you may use PLOS's Copyright Content Permission form.
Please respond directly to this email and provide any known details concerning your material's license terms and permissions required for reuse, even if you have not yet obtained copyright permissions or are unsure of your material's copyright compatibility. Once you have responded and addressed all other outstanding technical requirements, you may resubmit your manuscript within Editorial Manager.
Potential Copyright Issues:
- Please confirm that you are the photographer of Figure1B, or provide written permission from the photographer to publish the photo under our CC BY 4.0 license.

Photographer in Figure 1b and license are credited in figure legend. A link has been added. Image is in the public domain.

- Figure 1A; Please provide a direct link to the base layer of the map (i.e., the country or region border shape) and ensure this is also included in the figure legend; and provide a link to the terms of use / license information for the base layer image or shapefile. We cannot publish proprietary or copyrighted maps (e.g. Google Maps, Mapquest) and the terms of use for your map base layer must be compatible with our CC BY 4.0 license.
Note: if you created the map in a software program like R or ArcGIS, please locate and indicate the source of the basemap shapefile onto which data has been plotted.
If your map was obtained from a copyrighted source please amend the figure so that the base map used is from an openly available source. Alternatively, please provide explicit written permission from the copyright holder granting you the right to publish the material under our CC BY 4.0 license.
If you are unsure whether you can use a map or not, please do reach out and we will be able to help you. The following websites are good examples of where you can source open access or public domain maps:
* U.S. Geological Survey (USGS) - All maps are in the public domain. (http://www.usgs.gov)
* PlaniGlobe - All maps are published under a Creative Commons license so please cite u201cPlaniGlobe, http://www.planiglobe.com, CC BY 2.0u201d in the image credit after the caption. (http://www.planiglobe.com/?lang=enl)
* Natural Earth - All maps are public domain. (http://www.naturalearthdata.com/about/terms-of-use/).

We have added a link to the terms of use in the figure legend. The map is from Natural Earth and is in the public domain.

3) We note that your Data Availability Statement is currently as follows: "All code written in support of this publication, as well as simulation input files and generated data, will be made publicly available at Zenodo prior to publication. Code and data are currently available for

review at
Zenodo: https://zenodo.org/records/13355279?preview=1&token=eyJhbGciOiJIUzUxMiJ9.eyJpZCI6IjBjZGM2YjZjLWFiZmYtNGUwNy04OThkLWIzYjk1OGE0YTE4MSIsImRhdGEiOnt9LCJyYW5kb20iOiJkY2Q2NDQ3ZTg1MjgwZGYzYmU1MzhmODVhN2VmNDc5ZCJ9.3Mr4pDx6p7qjQN_4ZceIXubnriGIEDylTUmmhZSXpmz5dEl6FHldhtEVHuhyjOZH4VMpgH3DX1-W6I34Jv1GGQ.". Please confirm at this time whether or not your submission contains all raw data required to replicate the results of your study. Authors must share the “minimal data set” for their submission. PLOS defines the minimal data set to consist of the data required to replicate all study findings reported in the article, as well as related metadata and methods (https://journals.plos.org/plosone/s/data-availability#loc-minimal-data-set-definition).
For example, authors should submit the following data:
- The values behind the means, standard deviations and other measures reported;
- The values used to build graphs;
- The points extracted from images for analysis..
Authors do not need to submit their entire data set if only a portion of the data was used in the reported study.
If your submission does not contain these data, please either upload them as Supporting Information files or deposit them to a stable, public repository and provide us with the relevant URLs, DOIs, or accession numbers. For a list of recommended repositories, please see https://journals.plos.org/plosone/s/recommended-repositories.
If there are ethical or legal restrictions on sharing a de-identified data set, please explain them in detail (e.g., data contain potentially sensitive information, data are owned by a third-party organization, etc.) and who has imposed them (e.g., an ethics committee). Please also provide contact information for a data access committee, ethics committee, or other institutional body to which data requests may be sent. If data are owned by a third party, please indicate how others may request data access.

Yes, all raw data are included. We have updated our Data Availability Statement to state that the data release contains “data necessary to reproduce results.”

4) Please amend your detailed Financial Disclosure statement. This is published with the article. It must therefore be completed in full sentences and contain the exact wording you wish to be published. Please ensure that the funders and grant numbers match between the Financial Disclosure field and the Funding Information tab in your submission form. Note that the funders must be provided in the same order in both places as well.

- State the initials, alongside each funding source, of each author to receive each grant. For example: "This work was supported by the National Institutes of Health (####### to AM; ###### to CJ) and the National Science Foundation (###### to AM)."
- State what role the funders took in the study. If the funders had no role in your study, please state: "The funders had no role in study design, data collection and analysis, decision to publish, or preparation of the manuscript.".
If you did not receive any funding for this study, please simply state: u201cThe authors received no specific funding for this work.u201d

We have edited the financial disclosure statement as directed.
