# Supplementary material for: Host responses and viral traits interact to shape the impacts of climate warming on highly pathogenic avian influenza in migratory waterfowl
Source: PLoS Comput Biol. 2025 Oct 6;21(10):e1013451. doi: 10.1371/journal.pcbi.1013451 (PMC12513652; doi:10.1371/journal.pcbi.1013451)
Supplement: Attachment — Submitted filename: Review_Response.pdf [file pcbi.1013451.s025.pdf]

Editor: I believe that the remaining comments/questions from R1 can be addressed with additions to the text of the manuscript without more simulations, etc.. Please pay careful attention to ensure all of R1's comments are addressed in the revision.

Thank you for the opportunity to submit a revision. As noted in our correspondence with the Academic Editor Dr. Scarpino, it appears that Reviewer 1 was reading an older version of the manuscript. Below we respond to these comments, noting when these issues were already addressed in the first revision. We refer to the versions of the manuscript as R1 (the first revision) and R2 (the current submission).

Reviewer #1: This study investigates how climate change impacts the dynamics of highly pathogenic avian influenza (HPAI) in migratory waterfowl, focusing on the interplay between viral traits (e.g., transmission modes, environmental persistence) and host ecology (e.g., migration phenology). Using a mechanistic model, the authors simulate HPAI outbreaks under current and future climate scenarios, revealing that climate warming can either amplify or mitigate HPAI impacts depending on whether it affects viral decay rates or bird migration timing.

Major Concerns:

• The model assumes homogeneous mixing within sites, ignoring social structures (e.g., familial groups in geese). How might this bias transmission dynamics?

This comment is very similar to that from a reviewer in the first round of revisions. In R1, we added more information about the implications of non-homogeneous mixing to the discussion (Line 523-525). We also relaxed some of the assumptions related to homogeneous mixing by modeling multiple wintering sites, reducing the population size of modeled hosts, and reducing the contact transmission rate in the breeding season compared to the nonbreeding season.

• The study focuses on Pacific greater white-fronted geese. Would results generalize to other waterfowl, especially species with different migration patterns or tolerance to HPAI?

This study uses Pacific greater white-fronted geese as a focal system, but the use of sensitivity analyses and the general patterns of our results suggest that the qualitative patterns would generalize to other species. For example, we examine the sensitivity of model results to the HPAI-induced mortality rate, which is a proxy for tolerance of infection. Although other species might migrate to more sites or across different climates than the geese, the general characteristics of migration (i.e., most time spent at breeding and winter sites; breeding sites at higher/cooler latitudes) are consistent across waterfowl. In R1, we added a statement in the Introduction to justify the generality of migration phenology as the host response to climate change ("there is ample evidence of temperature-related changes to migration phenology over the last few decades," Line 93). The introduction also discusses the choice of greater white-fronted geese as a model system (Line 87-96) and migration responses to climate change more generally (Line 67-69). We have added the following text to the introduction in R2: "Although these geese are a particularly amenable system, the model could also generalize to other latitudinally migratory waterfowl" (Line 95-96).

• Only two SSP scenarios (2-4.5 and 3-7.0) are tested. Could more extreme or intermediate scenarios yield different insights?

Although we used these two intermediate scenarios in the original manuscript, we used only SSP 5-8.5 (an "extreme" scenario) in R1 because it provides the cleanest illustration of a "what-if" scenario for future climate change. Given the mechanisms in the model and our results, using less severe climate change scenarios would be very likely to produce the same patterns but smaller effect sizes. We continue using a single scenario in the current version (R2) but still acknowledge that our results might differ in magnitude under different climate scenarios (Line 541).

• The ranges for key parameters (e.g., direct transmission rate β2β2) are broad due to limited empirical data. How sensitive are conclusions to these uncertainties?

Our results show that the model is generally more sensitive to parameters with broad ranges, including the direct transmission rate. This comment is similar to that from a reviewer in the first round of revisions; in the revised manuscript (R1) we added a section in the discussion where we highlight that some parameters are difficult to define by nature (β), but that our results highlight others as potential targets for future empirical quantification (Line 484-489).

• Cross-immunity (ψ1,ψ2ψ1,ψ2) is fixed, but real-world variability in immunity across strains/hosts could alter dynamics. Was this explored?

Cross-immunity is fixed within a single parameterization (i.e., simulation) of the model and is identical across hosts in each parameterization but is still explored in sensitivity analysis of HPAI outcomes. We therefore incorporate variability in immunity across strains, but not across individual hosts. In R2, we have added cross-immunity to the Discussion where we highlight parameters that could vary among hosts: "the recovery rate, waning immunity rate, **and cross-immunity**, are based on ample data and thus have defined ranges (e.g., duration of immunity 3 months-2 years) but still vary among and within species and age classes" (Line 493; bold text added in R2).

• Lines 527–529 suggest environmental transmission leads to longer outbreaks, but results (e.g., Figure 3) show direct transmission drives higher peak prevalence. Clarify this discrepancy.

Peak prevalence measures the maximum infection prevalence achieved at any point during the time-series. In most cases, this peak occurs immediately after viral introduction. In contrast, outbreak duration and other long-term outcomes (i.e., mean infection prevalence) are not necessarily related to peak infection prevalence, in part because strains that cause high peak prevalence can produce enough immunity or mortality that they go extinct. Figures 4 and 5 in R1 and R2 show these results.

• The abstract states climate warming "increases HPAI impacts" (line 24), but results show opposing effects (e.g., reduced environmental persistence). Reconcile these statements.

This comment refers to text that was deleted in R1.

• How were migration timings validated against empirical data? Were interannual variations in migration considered?

Migration timing in the "baseline" models (i.e., LPAI and HPAI sensitivity analysis before climate change) are based on empirical data from GPS tracking of geese (Line 137-142, Figure 1C). In these models, we modeled migration as occurring over a range of dates. Because of the stochastic implementation of the model, this approach produced some

interannual variation in migration timing. However, each year in the model followed the same parameters (i.e., the range of dates was fixed); in this sense, interannual variation was not considered. Although this variation could potentially be important for AIV dynamics, we chose to exclude it to more clearly focus on viral, rather than host, parameters.

In the climate change simulations, migration timing was based on empirical relationships from literature. Although we explored timing-temperature relationships in the goose GPS data, our 6-year time-series was not long enough to reliably parameterize long-term trends in migration timing.

We have added to the Discussion that climate change could also alter the variance in migration timing (Line 545).

• The inverse relationship between decay rate and temperature sensitivity (line 214) is critical but relies on a single study (Handel et al.). Are there conflicting data?

The relationship between decay rate and temperature sensitivity was parameterized based on a single study (Handel et al. 2014; ref. 22), but this study conducted experiments using multiple influenza strains/subtypes (both HPAI and LPAI). The inverse relationship has been observed across multiple studies, one of which is cited in the manuscript (Handel et al. 2013; ref. 23). We are not aware of any conflicting evidence, nor is any presented in a more recent systematic review (Dalziel et al. 2016).

Dalziel, A. E., Delean, S., Heinrich, S., & Cassey, P. (2016). Persistence of low pathogenic influenza A virus in water: a systematic review and quantitative meta-analysis. *PLoS One*, 11(10), e0161929. https://doi.org/10.1371/journal.pone.0161929

• The 9-day advancement in spring migration (line 469) seems small relative to projected temperature increases. Is this biologically plausible?

This is a good point. Although this rate of advancement is based on a long-term study of geese in Alaska (Ward 2016), it is not entirely up to date, and there is substantial uncertainty in our understanding of how quickly migrants will adjust their timing to match climate change. We address this uncertainty in the discussion by acknowledging that migration might advance more than expected (Line 544).

• Microhabitat refugia (e.g., shaded wetlands) could buffer temperature effects. Were these accounted for?

Our estimates of environmental temperature were based on mean air temperature across the spatial extent of each site. We therefore potentially include microhabitat refugia, but it is likely that these small areas contribute relatively little to the mean temperature across large sites. We address microhabitats as a potential mechanism that could cause changes in our results ("It is possible that these results would differ under different climate scenarios or with the inclusion of fine-scale data on microhabitats (e.g., temperature stratification in water, freeze-thaw cycles, fine-scale differences in temperature between sun and shade), which could provide thermal refugia for hosts or virions [91]", Line 540-544).

• Figure 1: Missing panel labels (A–F) in the text layer. Ensure consistency with the caption.

We do not see this issue in R1.

• Figure 3: Y-axis labels for "Proportion of simulations" are cut off in panels A and B.
We do not see this issue in R1.

• Table S1: Clarify units for parameters like β2β2 (is it per day?). Include citations for parameter ranges.
Units and citations are already included in Table S1.

• Table S3: The note says β2=0β2=0 and ω2=0ω2=0 simulations were excluded, but why? This limits understanding of purely immunity-driven dynamics.
HPAI is unable to invade if neither direct transmission nor shedding (i.e., environmental transmission) are present in the model. We have edited the table legend to say that we excluded strains where "**both** $\beta_2 = 0$ and ω_2= 0" (bold text added in R2); this edit clarifies that we still included strains with direct transmission only and environmental transmission only.

• Typos:
o Line 38: "prior infection provides little immunityviruses" → missing space.
This comment refers to text that is not present in R1.

o Line 213: "imbeddinged": "embedded".
This comment refers to text that is not present in R1.

o Line 527: "replicated LPAI infection dynamics in geese [57], even while ignoring excluding": redundant phrasing.
This comment refers to text that is not present in R1.

o Line 24–25: "climate warming has opposing impacts... via viral persistence and bird migration phenology": rephrase for clarity (e.g., "via opposing effects on viral persistence and migration phenology").
This comment refers to text that is not present in R1.

o Line 348: "We modeled 10,000 potential combinations": "We simulated".
We have made this change.

Reviewer #2: I believe the paper is fit for publication and the authors have addressed all my concerns
Thank you.

Reviewer #3: I thought your changes were appropriate for my comments as well as the other reviewers.
Thank you.
